# Toward an Automated System for Nondestructive Estimation of Plant Biomass

**DOI:** 10.1002/pld3.70043

**Published:** 2025-03-19

**Authors:** Randall Kliman, Yuankai Huang, Ye Zhao, Yongsheng Chen

**Affiliations:** ^1^ School of Electrical and Computer Engineering Georgia Institute of Technology Atlanta Georgia USA; ^2^ School of Civil and Environmental Engineering Georgia Institute of Technology Atlanta Georgia USA; ^3^ Department of Civil Engineering University of Memphis Memphis Tennessee USA; ^4^ School of Mechanical Engineering Georgia Institute of Technology Atlanta Georgia USA

## Abstract

Accurate and nondestructive estimation of plant biomass is crucial for optimizing plant productivity, but existing methods are often expensive and require complex experimental setups. To address this challenge, we developed an automated system for estimating plant root and shoot biomass over the plant's lifecycle in hydroponic systems. This system employs a robotic arm and turntable to capture 40 images at equidistant angles around a hydroponically grown lettuce plant. These images are then processed into silhouettes and used in voxel‐based volumetric 3D reconstruction to produce detailed 3D models. We utilize a space carving method along with a raytracing‐based optical correction technique to create high‐accuracy reconstructions. Analysis of these models demonstrates that our system accurately reconstructs the plant root structure and provides precise measurements of root volume, which can be calibrated to indicate biomass.

## 1 Introduction

In the field of hydroponics, plant health analysis as well as the collection of various plant‐related metrics are important both in research labs and in industry. Specifically, the estimation of plant responses to nutrient availability is important for determining how well plants will grow in a specific environment. Knowledge of these factors helps inform how an environment can be controlled to optimize plant productivity and is the basis for fields such as Controlled Environment Agriculture (CEA).

Various studies have attempted to link plant biomass and nutrient uptake, but few have attempted to do so with high temporal resolution. The main method for analyzing root nutrient uptake has been through in situ soil studies as well as field‐size studies (Aguilar et al. 2021). Unfortunately, these studies have poor temporal resolution as soil cores must be taken at longer intervals because of their invasive nature. Infrequent measurement leads to a lower fidelity model of plant root growth which can lead to either overestimates or underestimates of plant nutrient application. Underapplication can lead to lower crop yields, and overapplication has been found to be toxic to local environments (Dodds et al. 2009). Higher temporal resolution allows for a more precise application of nutrients to plants and less environmental impact.

Historically, most root analysis methods are either invasive (Bucksch et al. 2014; Trachsel et al. 2011) or very expensive to remain noninvasive (van Dusschoten et al. 2016; Bodner et al. 2018). As referenced above, classical methods rely on taking soil cores and sieving out dirt to see how root structures progress. This method is inaccurate, as it only captures a small amount of the whole root structure and is also incredibly invasive, effectively mutilating a plant root and causing it to change its growth patterns as a result. Recently, a few studies have been able to break through this cost barrier using advances in 3D model generation through the use of turntable‐based 3D scanning methods (Clark et al. 2011; Fang, Yan, and Liao 2009). These studies have successfully shown that in situ methods can be used to create high‐quality 3D root models. Plant imaging still poses many challenges, however. Many voxel‐based methods are limited by hardware constraints such as memory or computational speed. Other methods focus only on the root or the shoot systems (Scharr et al. 2017). Very few methods consider the impact of optical distortion due to canister‐style systems (Masuda 2019). Our method is one of few that focuses on both root and shoot systems, addressing these challenges by providing a novel approach that combines flexible imaging and advanced reconstruction techniques to estimate plant biomass non‐destructively.

The main objective of this study is to develop an automated, low‐cost system for nondestructive estimation of plant root biomass throughout the plant's lifecycle in hydroponic systems. We achieve this by utilizing a robotic arm and turntable to capture images from multiple angles, allowing for comprehensive 3D reconstruction. Our key contributions include the novel use of a robotic arm for flexible imaging, which allows dynamic positioning of the camera to capture detailed images of root structures without the need for multiple fixed cameras or manual repositioning. Additionally, we develop customized 3D reconstruction techniques, including a voxel‐based volumetric reconstruction method using space carving and a raytracing‐based optical correction algorithm to address optical distortion due to refraction in the growth medium. By integrating root and shoot imaging, our approach provides a more comprehensive estimation of plant biomass than methods focusing solely on one system. Our system significantly improves the resolution at which these models can be generated and allows for biomass estimations with submillimeter resolution.

## 2 Approach

**FIGURE 1:**
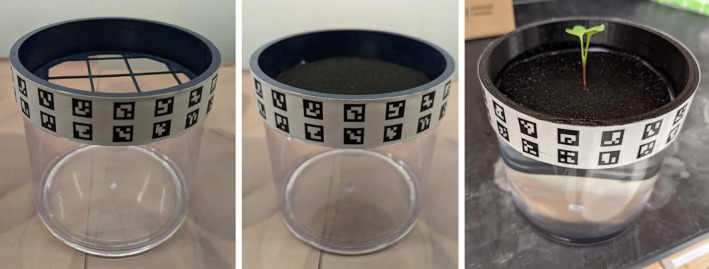
Left: Canister with net pot. Center: Canister and net pot with felt. Right: Canister with net pot, felt, and growing radish plant.

Plants are placed in a custom‐designed and 3D‐printed plant suspension device henceforth referred to as a “net pot”. These plants are then transferred to the net pot and held in place by two layers of black felt (Figure 1). The net pot is custom‐designed to suspend the root structure over a 6‐in. diameter by 6‐in.‐tall acrylic canister (Figure 1). This size was chosen to accommodate common root and shoot sizes for various plants. The net pot is designed to support plants, allow plants to grow freely without restriction, and provide sufficient aeration to the nutrient solution.

### 2.1 Imaging System

**FIGURE 2:**
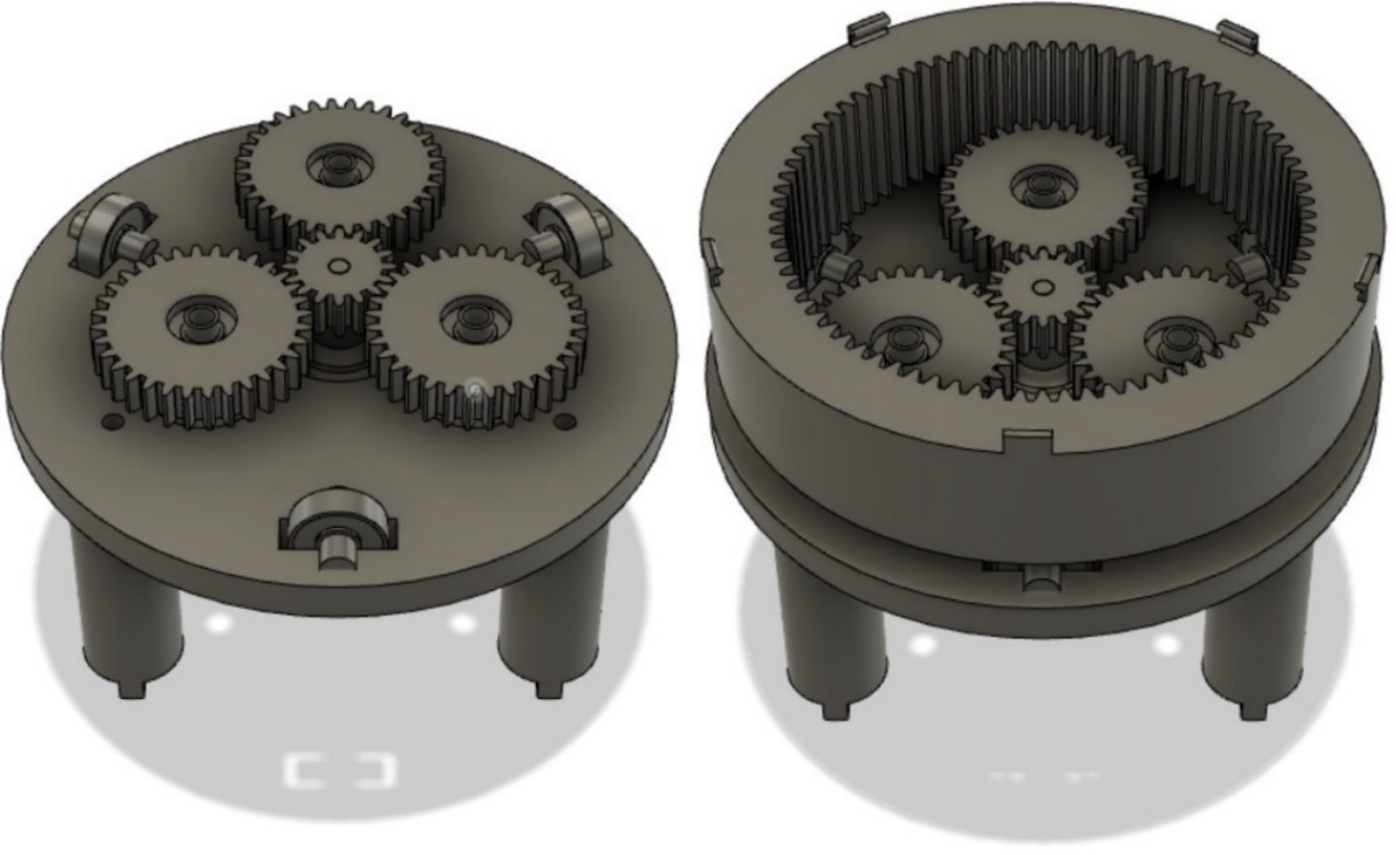
Turntable design. Left: Exposed turntable without ring gear. Right: Turntable with ring gear.

**FIGURE 3:**
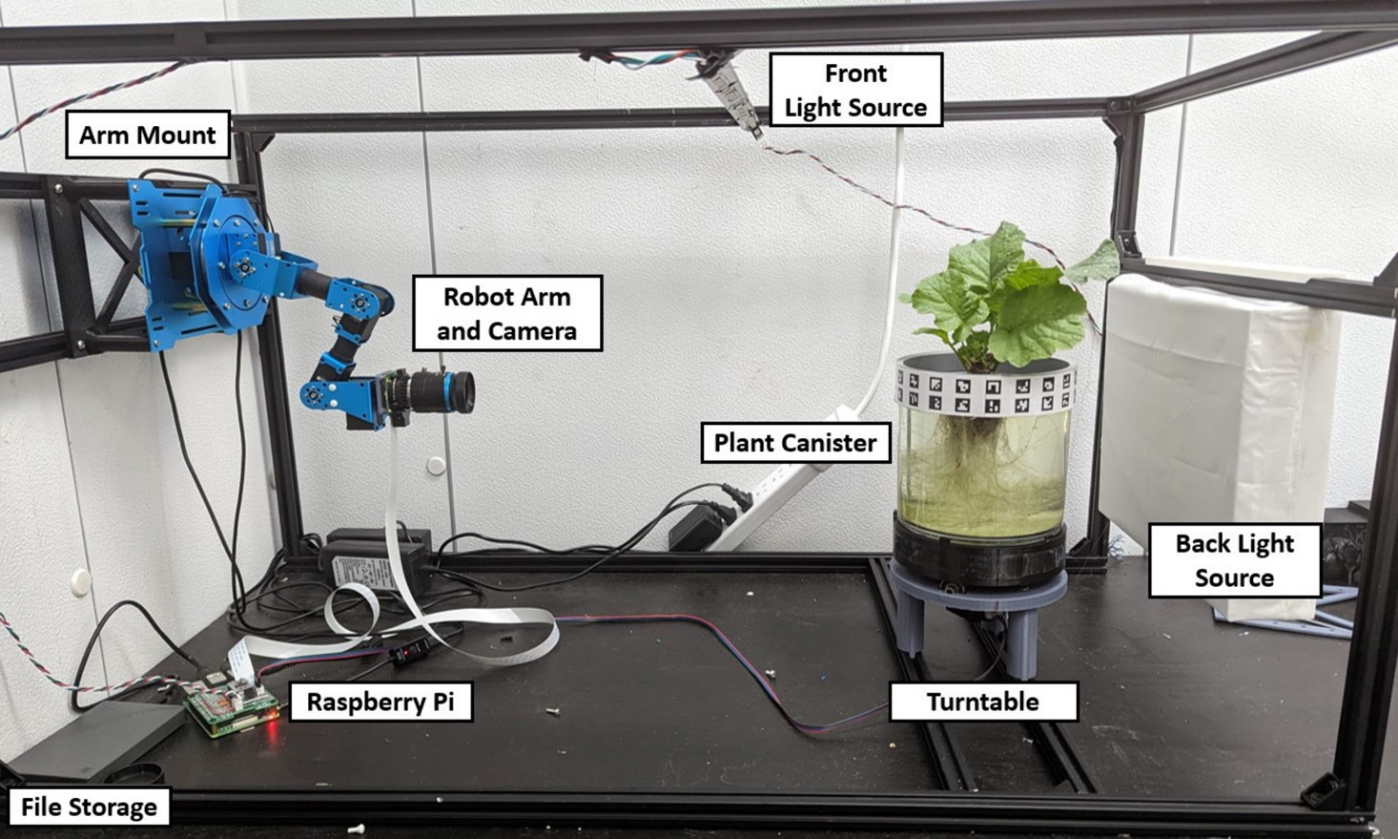
Imaging instrumentation. A Raspberry Pi HQ camera mounted on a robotic arm is pointed at a hydroponically grown plant mounted on a turntable, which is illuminated from behind by a custom LED lightboard.

**FIGURE 4:**
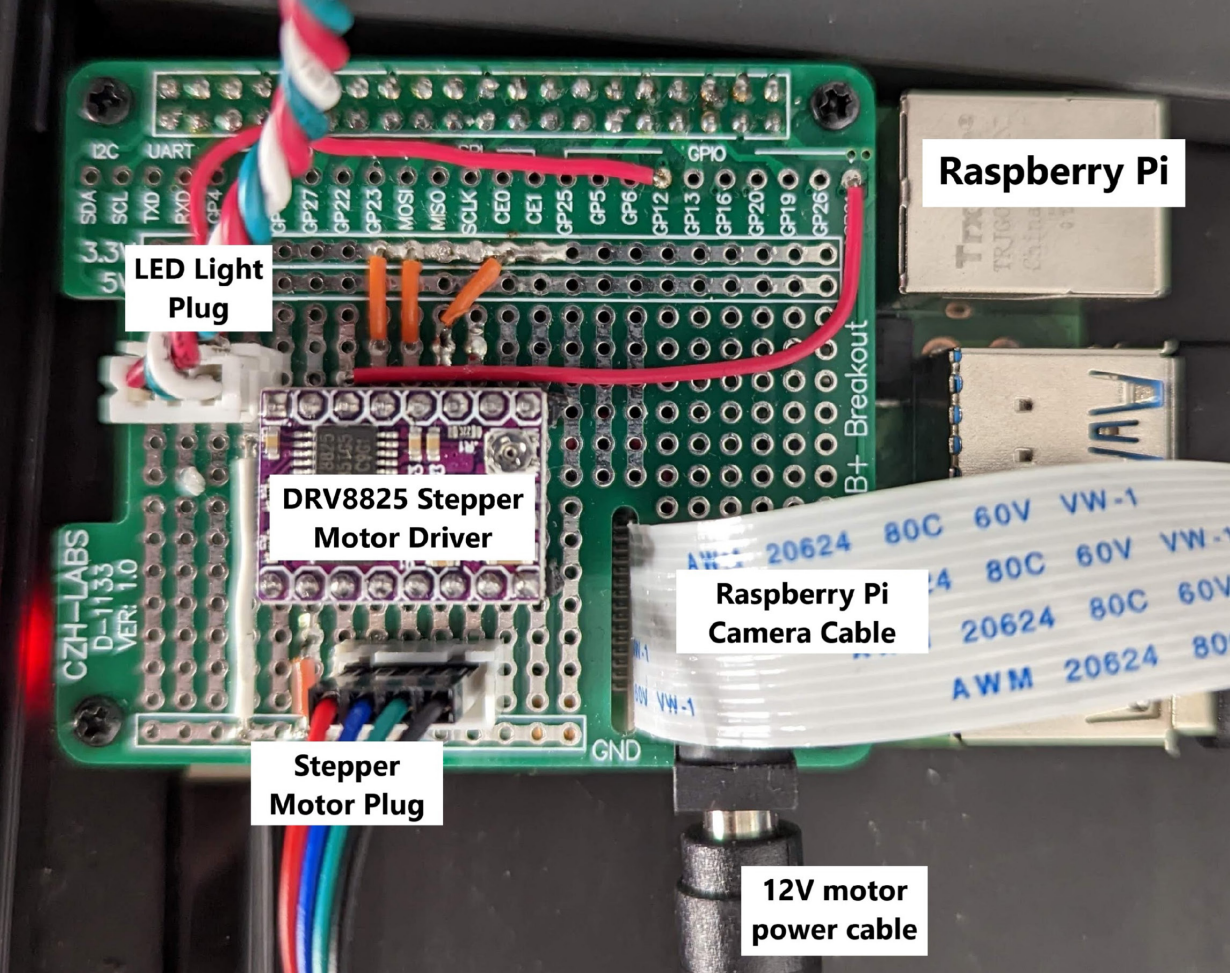
Raspberry Pi electronics connections. *Note:* Wiring is done on a protoboard shield that sits on top of the Raspberry Pi.

To image the lettuce plants, the canisters were placed on a custom‐designed and 3D‐printed turntable (Figure 2). This turntable is powered by one NEMA‐17 stepper motor (Applied Motion 2022) and an accompanying DRV8825 (Texas Instruments 2014) stepper motor driver. The imaging setup for this project is built into a 2020 aluminum extrusion box frame that allows for easy fixture and movement of experimental components. A key component of our imaging system is a robotic arm with a mounted camera, affixed to one end of the frame opposite the turntable (Figure 3). The camera system used is a Raspberry Pi High Quality Camera which has a 12.3‐megapixel sensor and can output RAW images. This camera was chosen for its high resolution, adjustable focus, and reasonable field of view. The camera system is connected to the Raspberry Pi using a Camera Serial Interface (CSI) cable and communicates with the Raspberry Pi over the CSI interfacing protocol. The primary reason for using the robotic arm is to facilitate the imaging of both root and shoot structures efficiently. The robotic arm allows for automated repositioning of the camera to capture images from optimal angles for both the roots and the shoots without manual adjustment or multiple cameras. This flexibility simplifies the imaging process and ensures consistent image quality throughout the experiment. For each image set, 80 images of the plant were taken: 40 images of the roots and 40 images of the shoots. These images are stored on a Raspberry Pi and then sent to an external computer for processing (Figure 4).

### 2.2 Camera Localization

Most plant imaging works use static cameras with known positions within their imaging space and take pictures of plant systems at known locations. This leads to high‐precision camera localization and little uncertainty about camera position relative to the subjects they capture. Unfortunately, because we use a camera mounted to the end effector of a low‐cost robotic arm, this same localization accuracy is not guaranteed. This problem is made worse by imperfections in the experimental design of the imaging frame and turntable.

**FIGURE 5:**
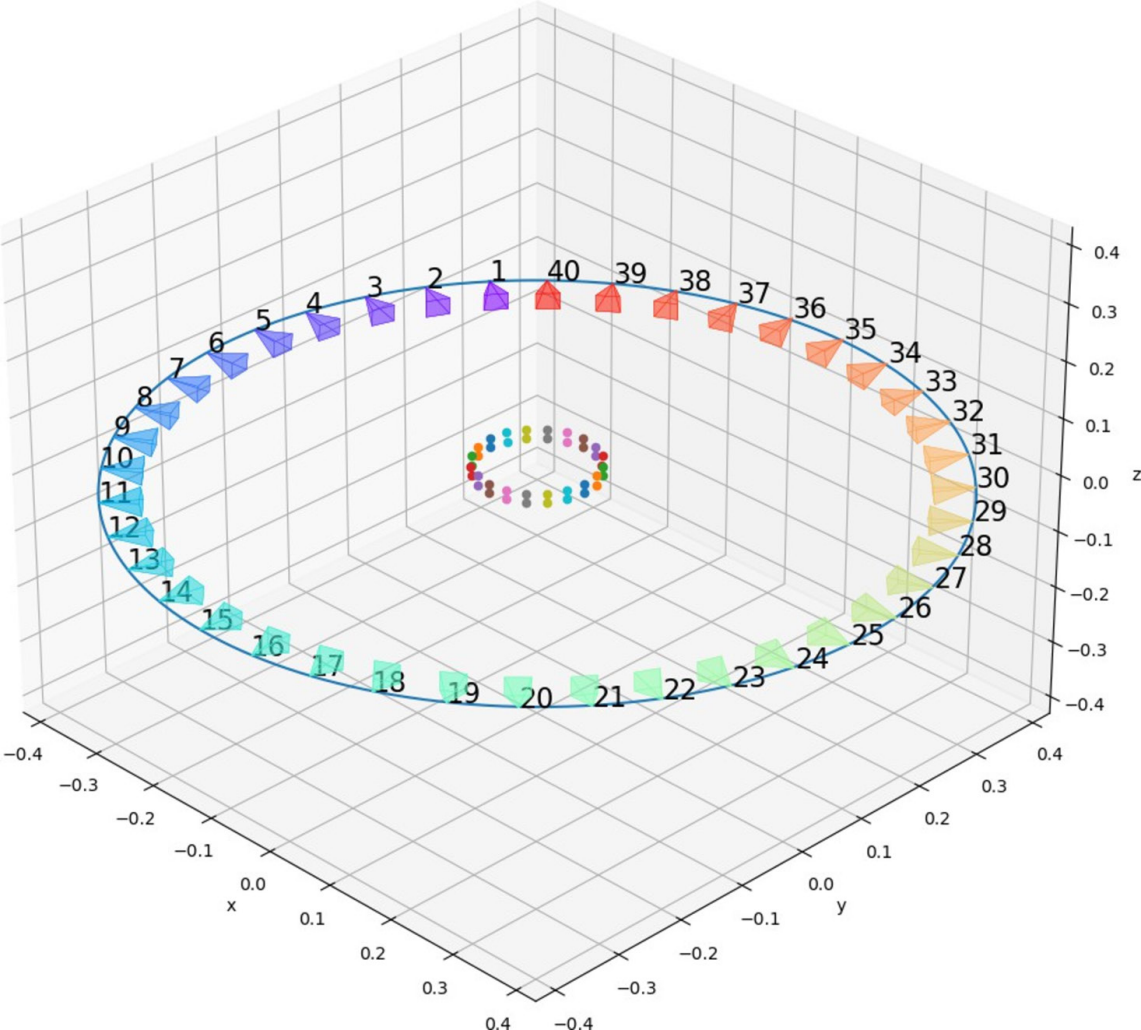
Camera pose estimations for each camera and the circle of best fit. The dots in the center are the positions of the fiducial markers.

To solve this issue, a strip of ArUco fiducial markers is affixed to the outside perimeter of each net pot (Figure 5). By knowing the positions of each marker in the real world as well as the position of each marker center in the captured images, we can localize the camera positions. This is usually done using a method called Perspective‐n‐Point (PnP), which has been conveniently implemented as a function in OpenCV. For PnP to work, four or more markers must be detected. The easiest way to do this is to make the marker strip two markers high so that many markers could be seen. This creates a sort of grid that can be easily detected by the detector algorithm. This grid can be easily printed and wrapped around the net pot, guaranteeing that the markers are at precise relative positions to each other. With the camera extrinsic generated, camera projection matrices can be calculated by multiplying the 3 × 4 camera intrinsic matrix with a 4 × 4 extrinsic matrix, leading to a 3 × 4 matrix.

### 2.3 3D Reconstruction

Many methods exist for 3D reconstruction, which include Structure from Motion (SfM) (Ullman 1979), Space Carving (Kutulakos and Seitz 1999), and Neural Radiance Fields (NeRF) (Mildenhall et al. 2020). Unfortunately, SfM and NeRF prove difficult to use because of the complex and sparse nature of root and shoot morphology. SfM and NeRF work by detecting and matching features between images. Root structures, however, do not have many identifiable features and small perturbations between images can make it difficult to match the few features they have. This causes issues when localizing the camera positions, causing very poor reconstruction results and even failure on the collected data sets. The Inverse Radon Transform is a foundational technique in computed tomography (CT) for reconstructing an object from its projections. It assumes parallel beam projections and requires precise control over projection angles and imaging conditions. However, applying IRT to our context poses several challenges, since plant root systems have complex, nonuniform shapes with concavities that are difficult to reconstruct accurately using IRT, which is better suited for objects with uniform properties. Additionally, IRT requires high‐quality, evenly distributed projection data. In our setup, achieving such precise control is challenging, and the silhouettes may contain noise due to imaging conditions. Thus, because SfM, NeRF, and IRT are unavailable to use, Space Carving is determined to be the most suitable method for 3D reconstruction. To implement this method, we develop a library in Python using extensive use of the OpenCV and NumPy packages. Space carving works by using silhouettes as masks to slice away from a virtual voxel volume. Given a set of silhouettes and camera positions, a visual hull can be created that should contain the object being scanned.

### 2.4 Silhouettes

Many methods exist for obtaining high‐quality silhouettes of root structures. Options include simple thresholding, hysteresis thresholding, and normalized intensity thresholding (Zheng et al. 2011). These methods work well for very thin root structures but fail to account for root vegetables such as radishes, potatoes, and carrots. Additionally, they require high‐quality lighting conditions and only work on images where the root system is the only thing visible. We propose a method for obtaining high‐quality silhouettes of root structures that involves combining the thin root preservation of hysteresis thresholding with the large structure detection of simple thresholding.

1. A global threshold is found using Otsu's method (Otsu 1979) and edges are detected using a Sobel filter.
2. The edges are thresholded using hysteresis to create a binary silhouette, which is then combined with a silhouette obtained through simple thresholding to fill large holes and preserve thin root structures.
3. Lastly, the resultant image is passed through an OpenCV open filter to remove as many pixel‐wide holes that may persist in the thin root structures.

**FIGURE 6:**
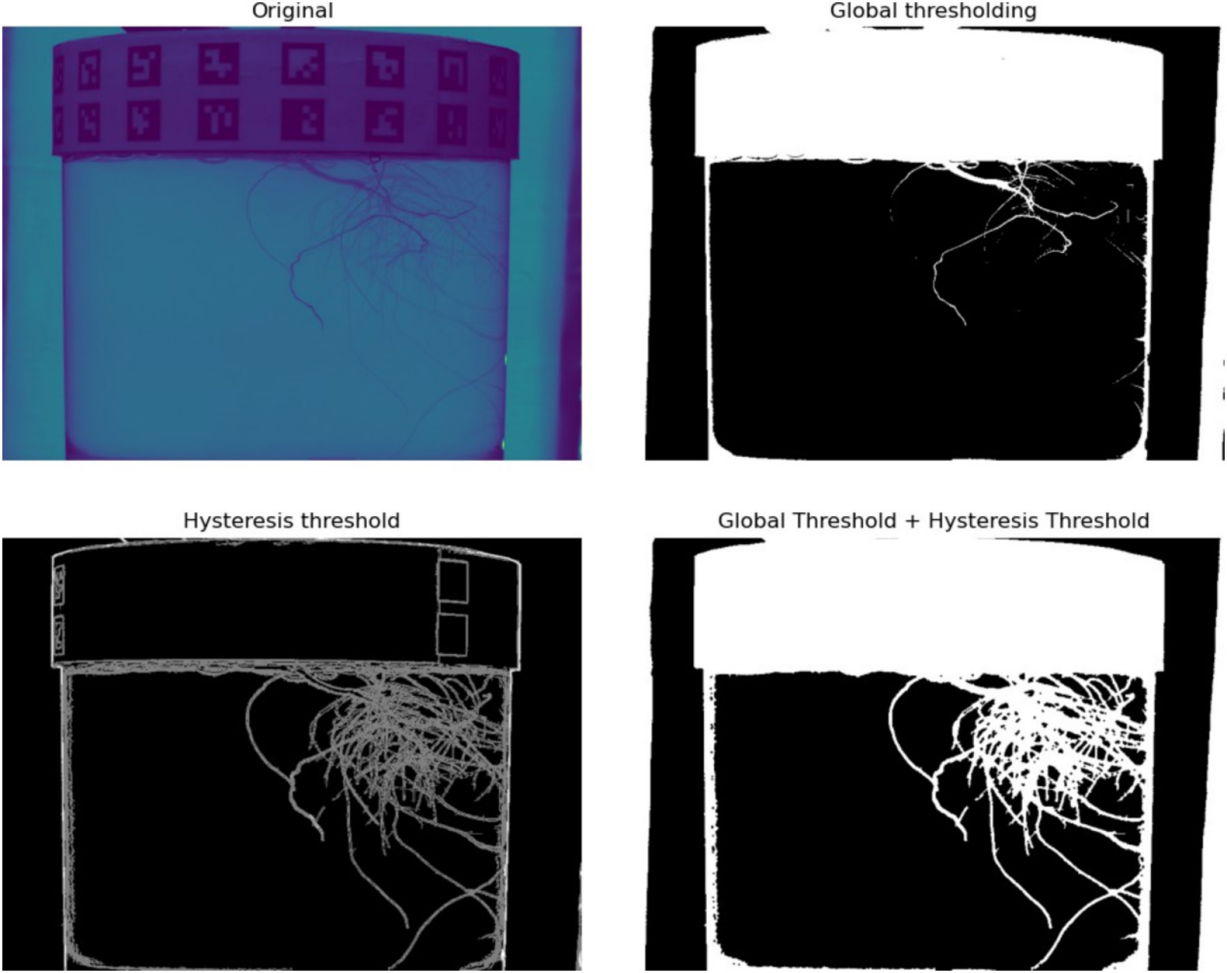
Silhouetting process to achieve full root silhouettes. *Note:* The whole canister is present, but using a raytracing technique, only the area inside the canister will be visible.

This often leads the root structures to be thicker than before; however, this is usually counteracted by the aggressive nature of the space carving method. Figure 6 shows how the combination of methods provides the best result.

### 2.5 Raytracing

One major challenge in the imaging of hydroponic systems is the distortion caused by refraction due to various growth media. In our experiment, we grow plants hydroponically in cylindrical acrylic canisters, which introduces significant optical distortion due to refraction at the interfaces between different media. There are two stages of refraction in our system: (1) Light rays traveling from the air into the acrylic canister refract at the outer surface because of the difference in refractive indices between air (*n* ≈ 1.00) and acrylic (*n* ≈ 1.49); (2) light rays refract again at the inner surface of the canister when passing from acrylic into the nutrient solution (water with dissolved nutrients, *n* ≈ 1.33). These two stages of refraction cause significant optical distortion, making objects inside the canister appear compressed or shifted from their true positions. This distortion affects both the apparent size and shape of the root structures, leading to inaccuracies in camera pose estimation and 3D reconstruction if not properly corrected.

**FIGURE 7:**
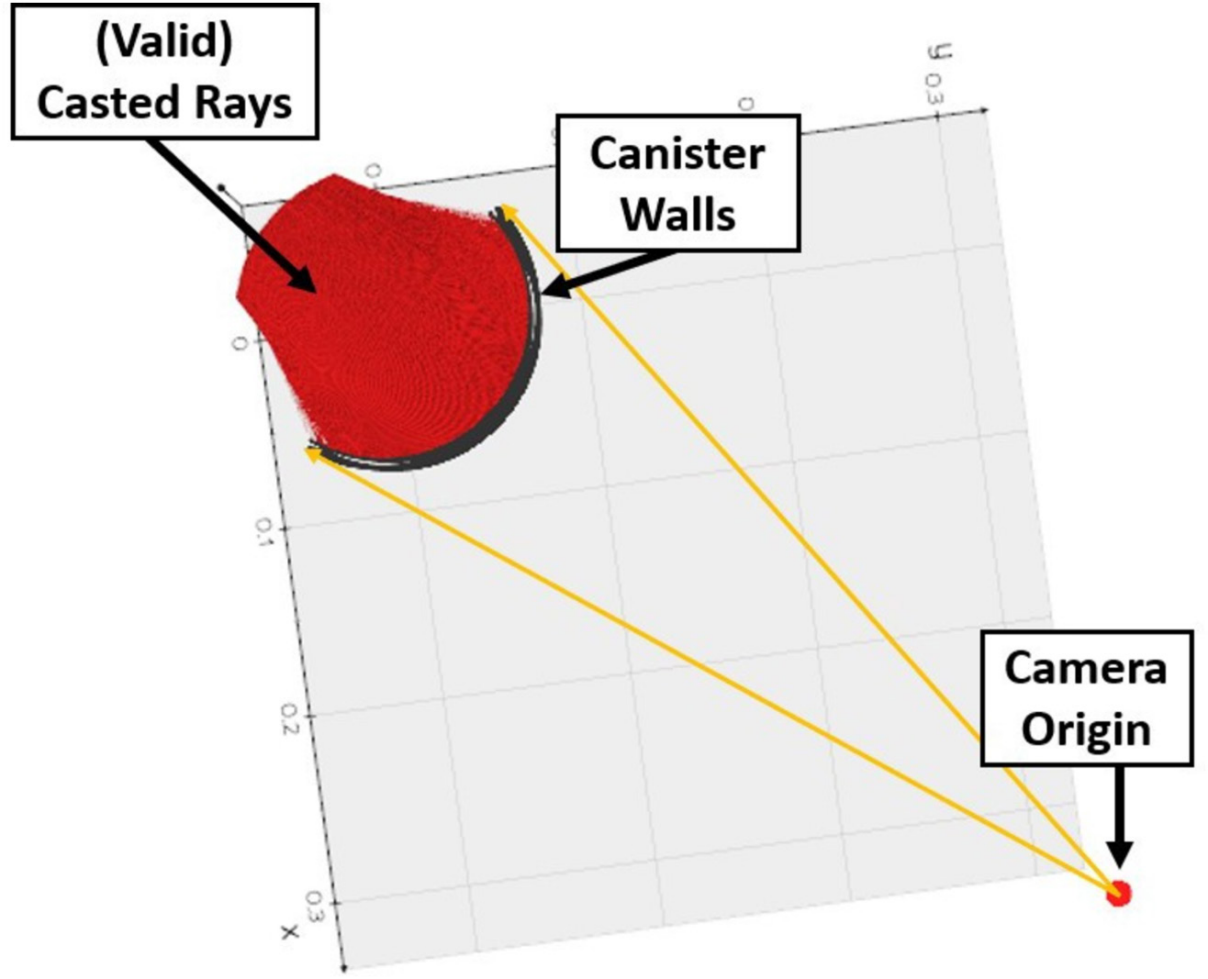
Raytracing visualization. Rays leave the camera origin and are cast (yellow lines) toward the canister walls. Rays refract through these walls and end up as the red rays.

To address this issue, we developed a raytracing algorithm based on previous research and used the previously calculated camera extrinsic for each view. We cast 480,000 (800 × 600) rays from the camera at a simulated cylinder with the same dimensions as the growth canister, and after these rays collided and refracted on both the inside and outside surfaces of the canister, a set of points and their resulting ray vectors are generated on the inside surface of the cylinder. Figure 7 visualizes this process. Optical distortion affects the estimation of root volume by altering the apparent dimensions of the root structures. The refraction causes the roots to appear smaller or differently shaped than they actually are, which can lead to underestimation or overestimation of the root volume when reconstructing the 3D model. Without correction, these distortions can significantly compromise the accuracy of biomass estimations.

For each voxel at every view, we cast these rays toward virtual voxels and checked for intersection. Using a 3x800x600 ray tensor with a 3D vector as the first dimension and the remaining dimensions as the shape of our images, we created a binary mask indicating whether the ray intersected a voxel. This mask can be applied to our original silhouette using a bitwise AND operation to determine the proportion of each voxel that is visible, allowing for a more accurate estimation of voxel membership in the model. By applying this correction, we effectively mitigate the distortion caused by refraction, aligning the apparent positions of the root structures with their true positions. This correction improves the accuracy of camera pose estimation and the subsequent 3D reconstruction of the root system. To speed up this process, we developed a raytracing shader that was applied to an image for each voxel and was parallelized on a GPU using the Python package CuPy (Okuta et al. 2017).

### 2.6 Volume Computation

After reconstructing the 3D model of the plant roots using the octree‐based space carving method, we computed the volume by summing the volumes of all occupied voxels in the final octree structure. Each voxel represents a small cube in 3D space, and its volume is determined by the cube of its side length. The total root volume *V*_total_ is calculated as

$$V_{\text{total}}=N\times s^{3}$$

where *N* is the total number of occupied voxels and *s* is the side length of a voxel, determined by the resolution of the octree. This method allows us to estimate the volume of the root system based on the spatial occupancy of the voxels in the reconstructed model.

### 2.7 Octree Space Carving

In order to perform efficient space carving, we used a method based on Scharr's octree‐based silhouette space carving approach (Scharr et al. 2017). The octree model has several advantages, including its memory efficiency for storing large amounts of spatial data and its ability to store information about the size of each voxel, which is useful for occupancy tests. Scharr's method involves creating a large voxel with a specified bounding box, projecting it onto the silhouette, and checking how much of the silhouette it contains. If the voxel contains all of the silhouette, it is marked as part of the object. If it contains some but not all of the silhouette, it is split into eight voxels. Otherwise, it is marked as empty. In our algorithm, we used the raytracing method described previously to determine voxel membership and continue splitting voxels until a certain desired resolution is reached. We applied this process from each imaged angle until the root structure emerges, using the “mark‐and‐refine” method to project and mark all voxels for each view at each step and creating a queue for the voxels to be checked. We chose this method over a static grid of points because of its superior memory and speed performance compared with a static voxel grid with a predetermined number of voxel points.

## 3 Experimental Methods

### 3.1 Algorithmic Validation

**FIGURE 8:**
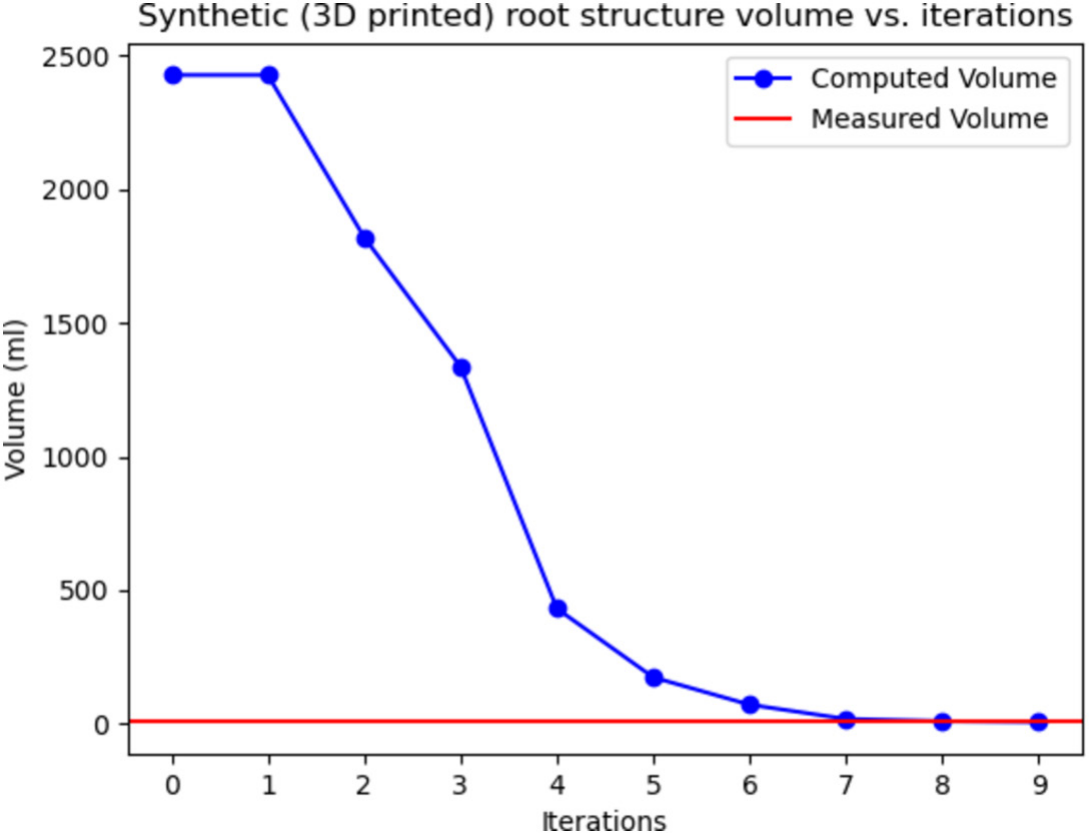
Top: Estimated volume of a synthetic (3D printed) root structure.

First, we sought to validate that the model could accurately reconstruct plant volume. This was done in two ways. The first validation was done by printing a set of cubes in PLA ranging from 50 × 50 × 50 mm (125 mL) to 125 × 125 × 125 mm (1953.125 mL). The algorithm performed well, reconstructing each to a roughly 89% accuracy. We also validated our model by 3D printing synthetic root structures in solid PLA and comparing their measured volume using water displacement with their calculated volume using our method. We measured the synthetic root structure to be 8 mL and calculated the volume to be 7.13 mL. These measurements were very close, and the error can be attributed to poor camera pose estimation as well as aggressive voxel culling. Much of the synthetic root's volume is in its top, which has been clipped slightly during reconstruction. This is caused by poor camera pose estimation as well as strange camera angles. However, the roots looked qualitatively correct, meaning that the raytracing method was effective in correcting for optical distortion. Figure 8 illustrates how the space carving algorithm approached the volume of the synthetic root given enough iterations.

To process the image sets efficiently, we limited the voxel resolution in our 3D reconstruction algorithm to approximately 0.27 mm. This resolution was selected because of computational constraints, as higher resolutions would require significantly more memory and processing time than was available on our hardware. Although a finer voxel size could capture more detailed features of the root structures—such as thin roots and root hairs—the chosen resolution provides a balance between reconstruction quality and computational feasibility.

### 3.2 Experimental Setup

Next, in a proof‐of‐concept experiment, we aimed to evaluate the performance of our system by growing a large number of plants and creating a comprehensive, longitudinal dataset that demonstrated the algorithm's ability to handle a range of plant sizes and topologies, from small and simple to large and complex. This experiment also served to calibrate our non‐destructive system by using the data to approximate the density of roots and shoots, enabling us to measure biomass in subsequent experiments without destroying the plants. We conducted a longitudinal study of 18 radish plants over 30 days, imaging three plants every 5 days using a triplicate system to capture variability and get a better understanding of a plant's appearance at different points in its growing cycle (Figure S1).

Plants were first germinated for 7 days in a rock‐wool medium before being removed from the medium and transferred to the canisters. Plants were placed in the net pot and positioned in the center of the canister using two pieces of felt for support. A hole was cut in the center of the circular felt pieces where the plant's roots were carefully positioned through the hole with the roots on the bottom side of the felt and the shoot on the top side of the felt. The canisters were filled with a nutrient solution (Table S1), and the net pot was placed on top of the canister. The canisters were then placed on a shelf unit that contained a grow light directly above them. The grow lights were set on a timer to be on for 16 h during the day and off for 8 h at night. Tin foil was wrapped around the canisters to mitigate algae production. At 5‐day intervals, a set of three plant canisters were removed from the shelf, placed onto the turntable, and imaged using the camera technique. After imaging, the plants were cut to separate roots and shoots, and their respective wet weights were measured. Next, the plants were dried in an oven for 2 days after which their dry masses were measured. Lastly, the pictures taken by the imaging system were sent through the 3D reconstruction algorithm to determine the root volumes.

## 4 Experimental Results

**FIGURE 9:**
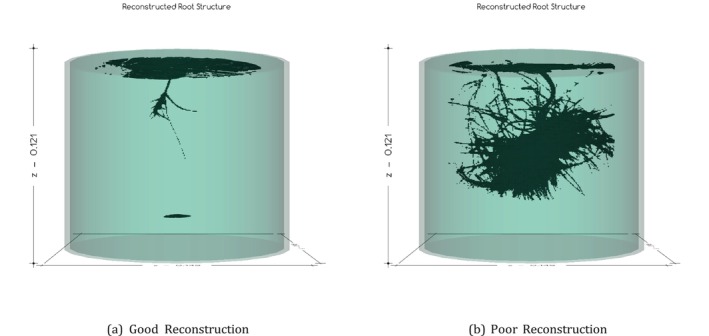
Qualitative reconstructive comparison. (a) A good reconstruction. Roots are easy to see, and extraneous voxels are clearly not part of the root structure. (b) A poor reconstruction. Blobbing during silhouetting causes huge reconstructive masses and volumetric overestimations.

We applied our 3D reconstruction algorithm to the image sets of all 18 plants, running the process on a super‐computing cluster to minimize interruption due to the long run time. Because of the processing time, the resolution was limited to approximately 0.27 mm instead of the theoretically possible 70 μm. The quality of the resulting 3D reconstructions and volumetric estimates varied, with six considered “good” reconstructions based on how closely the reconstructed root resembled the original root structure and 12 considered “poor” reconstructions due to the high complexity of the root structure making it difficult for the silhouetting algorithm to accurately identify specific roots. These errors resulted in large blobs of roots in the 3D reconstructions. Figure 9 compares a set of “good” and “poor” reconstructions.

As expected, the volume of plant roots tends to increase over time. However, the calculated volumes become very large and inconsistent in the latter half of the experiment and result in an overestimation of the volume. To estimate the density of the roots, we divide the calculated volume by the measured wet mass of the roots. Combining all the data, we find that the average plant density is 0.034 g/mL. We can also track how the density of the plants changes over time. Based on the wet and dry mass data, we expect that root density will decrease over time because the percentage of water in the roots increases over time. Additionally, we know that plant matter sinks in water, meaning it is denser than water. So, an increase in water mass will lead to a decrease in density. Table 1 shows how this density changes over time. However, the densities calculated in Table 1 show that the system does not provide accurate volumetric estimations for all types of plants. Although the structure of the roots is often qualitatively correct based on human comparison, the computed volumes are not accurate. This is because extraneous voxels, aggressive voxel culling, and optical distortion cause high variability in the volume calculation, resulting in an overestimation of the volume. This overestimation leads to a root density that is much lower than the density of water, which is incorrect for two main reasons. First, root mass has been measured to be roughly 96% water by mass, so we should expect the root density to be relatively close to the density of water. Instead, the computed densities are closer to 10% of water's density, which has been calculated to be roughly 0.997 g/mL. Second, roots have been observed to sink in water, meaning that they are more dense than water. However, the computed densities of the root structures are much less than the density of water.

**TABLE 1:** Measured root volumes, masses, and densities. 3D reconstruction efforts on Roots 17 and 18 failed; thus, there are no estimates of volume or density for these measurements.

Root #	Days elapsed	Volume (mL)	Mass (g)	Density (g/mL)	Root % water
1	5	1.790	0.035	0.019	90.52%
2	5	2.302	0.059	0.025	95.04%
3	5	5.940	0.063	0.011	94.24%
4	10	1.000	0.119	0.119	97.06%
5	10	6.370	0.240	0.038	96.42%
6	10	3.563	0.262	0.073	97.36%
7	15	7.704	0.333	0.043	97.45%
8	15	3.352	0.148	0.044	98.17%
9	15	6.655	0.187	0.028	96.52%
10	20	73.420	0.956	0.013	96.82%
11	20	69.090	0.929	0.013	96.35%
12	20	31.250	0.590	0.019	96.97%
13	25	116.740	1.772	0.015	97.97%
14	25	17.290	0.696	0.040	98.09%
15	25	46.020	1.149	0.025	97.53%
16	30	68.230	1.180	0.017	97.79%
17	30	—	0.3414	—	97.57%
18	30	—	1.7325	—	97.71%
Density of water:				0.997	

We also observed that the reconstruction did not capture the full radius of the growth canister, likely due to optical distortion caused by refraction in the acrylic canisters. This prevented objects at the edges of the canister from being fully visible to the camera and resulted in a lack of photoconsistency, causing these voxels to be removed. Despite these limitations, the 3D reconstructions allowed us to quantitatively measure the volume of the roots, which provided valuable information on the growth and development of the plants. Overall, although the underlying algorithm may work for synthetic objects, there are a number of variables in real root structures that cause the methodology to produce invalid results. These variables include extraneous voxels, aggressive voxel culling, and optical distortion, which lead to an overestimation of the volume and an incorrect calculation of the root density.

Figure S2 shows how much time each iteration adds to the space carving for the 18 plants grown. The graph shows that the data follow a roughly exponential scale, reaching a timing complexity of around 5^*n*^ where *n* is the number of iterations. Thus, it made sense to limit the voxel size, as an additional iteration step would increase our resolution to approximately 0.13 mm but would lead to a 5× increase in computational time. Additionally, more mature root systems cannot benefit from the computational gains of the octree data structure because of their spread‐out nature and higher volume. For example, root structures in the early stages of development can be processed to 0.27 mm in only 25 min, but more mature root structures take nearly 24 h to process. Increasing the resolution to 0.13 mm would increase processing time to 125 min for a small root system and up to 5 days for a mature root system.

## 5 Discussion

The instrumentation for this system performed to expectations, allowing for accurate control of the robot and micron‐level accuracy in camera localization. However, the robot's kinematic model limited its ability to correct orientation or move sideways. Additionally, a robot arm may not have been necessary for root imaging, but its inclusion provides flexibility for multiple camera angles and the future ability to swap camera types for multispectral imaging. The turntable and lighting systems also performed adequately, allowing for high‐quality plant root and shoot images to be taken from a variety of different angles.

The 3D reconstruction algorithm used in this work is generally accurate but struggles on real‐world datasets because of several issues that cause inaccuracies. One main issue is distortion, which occurs when voxels near the edges of the canisters are not visible, leading to significant inaccuracy in volumetric estimation. To address this issue, the use of an “undistortion tank” was considered, where canisters are submerged in a water‐filled rectangular tank so that light reaching the camera does not refract around a curved surface. However, this solution had several problems, such as the potential for water damage to fiducial markers and the need for specific camera angles to ensure that both the root structures and the fiducial markers could be seen. Additionally, the rectangular tank can distort images slightly by magnifying them, requiring raytracing to correct for this magnification in software. These are minor issues in retrospect, and an undistortion tank may still prove useful in future works.

Another issue is extraneous voxels, which can be partially addressed by changing the camera angle so that the top and bottom surfaces of the canisters are not visible, reducing the number of voxels that need to be processed. However, extraneous voxels may still be present, and a user interface for voxel removal may be useful. The toolbox vedo (Musy et al. 2022) may be able to support mouse selections for this purpose.

An inherent limitation of our optical imaging method is the issue of occlusion, particularly when dealing with complex and dense root systems. Occlusion occurs when parts of the root structure block others from the camera's view, leading to incomplete or less accurate reconstructions. Although X‐ray CT scans can effectively overcome occlusion and provide high‐accuracy 3D reconstructions, they are expensive and less accessible and involve potential safety concerns due to radiation exposure. Our approach prioritizes practicality and cost‐effectiveness, aiming to provide a nondestructive biomass estimation technique that is accessible for broader applications. Future work may focus on mitigating occlusion effects by increasing the number of imaging angles or incorporating advanced computational methods to improve reconstruction accuracy despite occlusions.

The most important issue to address is aggressive voxel culling, which occurs when misalignment in camera positions causes sections of a visual hull to be wrongfully removed, particularly in the case of thin, wire‐like objects. To fix this issue, we attempted a regularized visual hull method from (Zheng et al. 2011). However, this method results in an overestimate of computed root volume. Further research is needed to find a better solution for this issue, as it is important for accurately estimating plant biomass.

We can also quantify the temporal performance of our algorithm. Our proposed methodology has a high time to model completion, making it less suitable for high‐throughput applications. To reduce the time required, we lowered the resolution from 70 μm to 1/3 of a millimeter. However, the time complexity of the model grows exponentially with the number of voxels, resulting in run times that are significantly longer than other approaches in the literature. Although our algorithm is slower than those using a fixed grid, it is significantly faster than using a traditional fixed voxel grid. Raytracing also proved to be a significant runtime bottleneck for this research, and even running the code on the GPU still caused the reconstruction algorithm to run slowly.

Compared with traditional methods for plant biomass estimation, which often involve destructive sampling and labor‐intensive processes, our nondestructive imaging approach provides a practical alternative with reasonable accuracy. Although advanced imaging techniques like X‐ray CT and MRI offer high‐resolution reconstructions and can mitigate issues like occlusion, they are costly and less accessible for widespread use. Recent optical imaging methods have improved nondestructive analysis but still face challenges with complex root structures and optical distortions. Our method strikes a balance between cost, accessibility, and performance, offering improvements over traditional destructive techniques and serving as a feasible option compared to expensive imaging technologies.

## 6 Conclusions and Future Work

We developed a method for creating accurate and memory‐efficient 3D models of plant root and leaf structures. Our model outperformed a previous method in resolution and was able to image plant root systems. We used a robot arm to create a 3D scanning tool that can generate high‐resolution images of plant roots without causing damage. Our method was able to accurately reconstruct simple plant roots but had difficulty with more complex structures. However, it was able to create a digital twin of a root structure using accurate methods and the reconstructed plants were dimensionally accurate. Our methodology is not entirely novel, but it combines existing methods to create a holistic approach for plant root reconstruction. Although it did not produce accurate results on the experimental dataset, it requires only a few modifications to work properly. The main issues to overcome are optical distortion and aggressive voxel culling. Despite these challenges, the algorithm has the potential to be a very accurate plant biomass estimator.

The computational methodology used in this project resulted in accurate models for each plant, but it has significant limitations. It was written in Python with extensive use of the NumPy package, which is slower than other programming languages. To improve runtime, GPU acceleration was used to make the raytracing faster. Future work could focus on improving the runtime of the ray tracer, as it is slower than the method proposed in Scharr et al. (2017) despite being more accurate. Additionally, the algorithm could benefit from being rewritten in a faster language like C++.

Although this system can create high‐resolution models of plant root structures, it is not suitable for scaling to the size required by the hydroponics industry. However, it may have potential as a tool for fine‐tuning nutrient solutions for large‐scale systems in a research and development setting. There are several directions for future research, including the skeletonization of root structures and the modification of the existing code to model plant shoot structures. The system was also intended to study how plants respond to changes in their environment, specifically nutrient changes, but time constraints limited this work.

Although we collected shoot images during our experiments, the analysis and reconstruction of the shoot system were considered beyond the scope of this research. Our primary goal was to address the significant challenges in root biomass estimation. Future work may involve analyzing the shoot images to provide a comprehensive assessment of total plant biomass and to further compare the differences in imaging conditions between roots and shoots.

Despite its limitations, the system has the potential for a wide range of experimental applications in the future, including studying plant interactions and their impact on the environment. Coplanting has numerous environmental benefits, but there are few methods for modeling these plant root interactions in 2D or 3D (Cabal et al. 2020). Our platform also allows for the creation of longitudinal data sets on plant roots interaction, which could provide insight into how plants compete and cooperate while growing together. Other possible experiments include examining the relationship between the microbiome around roots and their effect on root development and plant productivity (Wu et al. 2021; Schenk 2006).

## Author Contributions

**Experimental concept and design:** Randall Kliman and Yongsheng Chen; **Performed experiments:** Randall Kliman and Yuankai Huang; **Data analysis:** Randall Kliman, Yuankai Huang, Ye Zhao, and Yongsheng Chen; **Paper draft:** Randall Kliman; All authors have contributed significantly to data analysis, paper writing and editing, and revision.

## Conflicts of Interest

The authors declare no conflicts of interest.

## Peer Review

The peer review history for this article is available in the Supporting Information for this article.

## Supporting information


**Data S1.** Peer Review

**Figure S1:** 9 of the 18 plants grown for the experiment. Plants are labeled with their plant type, planting date, and initial volume. A set of grow lights was hung above the plants to assist in growth.
**Figure S2:** Time elapsed vs. Space Carving iteration number for our dataset. Timing is plotted on a logarithmic scale.
**Table S1:** Standard nutrient solution for plant growth. This combination of volume and mass ratios was specifically designed for a 6 L container. For some compounds, a pre‐mixed water concentrate was not available, so its corresponding solid salt form was used instead. Also, compounds H_3_BO_3_, MnSO_4_·H_2_O, Na_2_MoO_4_·2H_2_O, CuSO_4_·5H_2_O, and ZnSO_4_·7H_2_O were mixed together as a single mix of micronutrients.

## Data Availability

All data used in this study are available from the corresponding author upon reasonable request.
