# Supplementary material for: Toward an Automated System for Nondestructive Estimation of Plant Biomass
Source: Plant Direct. 2025 Mar 19;9(3):e70043. doi: 10.1002/pld3.70043 (PMC11920584; doi:10.1002/pld3.70043)
Supplement: Supplementary file 2 — Figure S1: 9 of the 18 plants grown for the experiment. Plants are labeled with their plant type, planting date, and initial volume. A set of grow lights was hung above the plants to assist in growth. Figure S2: Time elapsed vs. Space Carving iteration number for our dataset. Timing is plotted on a logarithmic scale. Table S1: Standard nutrient solution for plant growth. This combination of volume and mass ratios was specifically designed for a 6 L container. For some compounds, a pre‐mixed water concentrate was not available, so its corresponding solid salt form was used instead. Also, compounds H3BO3, MnSO4·H2O, Na2MoO4·2H2O, CuSO4·5H2O, and ZnSO4·7H2O were mixed together as a single mix of micronutrients. [file PLD3-9-e70043-s002.docx]

Supporting Information for

**Towards an Automated System for Nondestructive Estimation of Plant Biomass**

Randall Kliman^1^, Yuankai Huang^2, 4^, Ye Zhao^3^, Yongsheng Chen^2∗^

^1^School of Electrical and Computer Engineering, Georgia Institute of Technology, Atlanta, Georgia 30332, United States

^2^School of Civil and Environmental Engineering, Georgia Institute of Technology, Atlanta, Georgia 30332, United States

^3^School of Mechanical Engineering, Georgia Institute of Technology, Atlanta, Georgia 30332, United States

^4^Department of Civil Engineering, University of Memphis, Memphis, Tennessee 38152, United States

(*corresponding author: Yongsheng Chen, Email: yongsheng.chen@ce.gatech.edu, Phone: 4048943089)


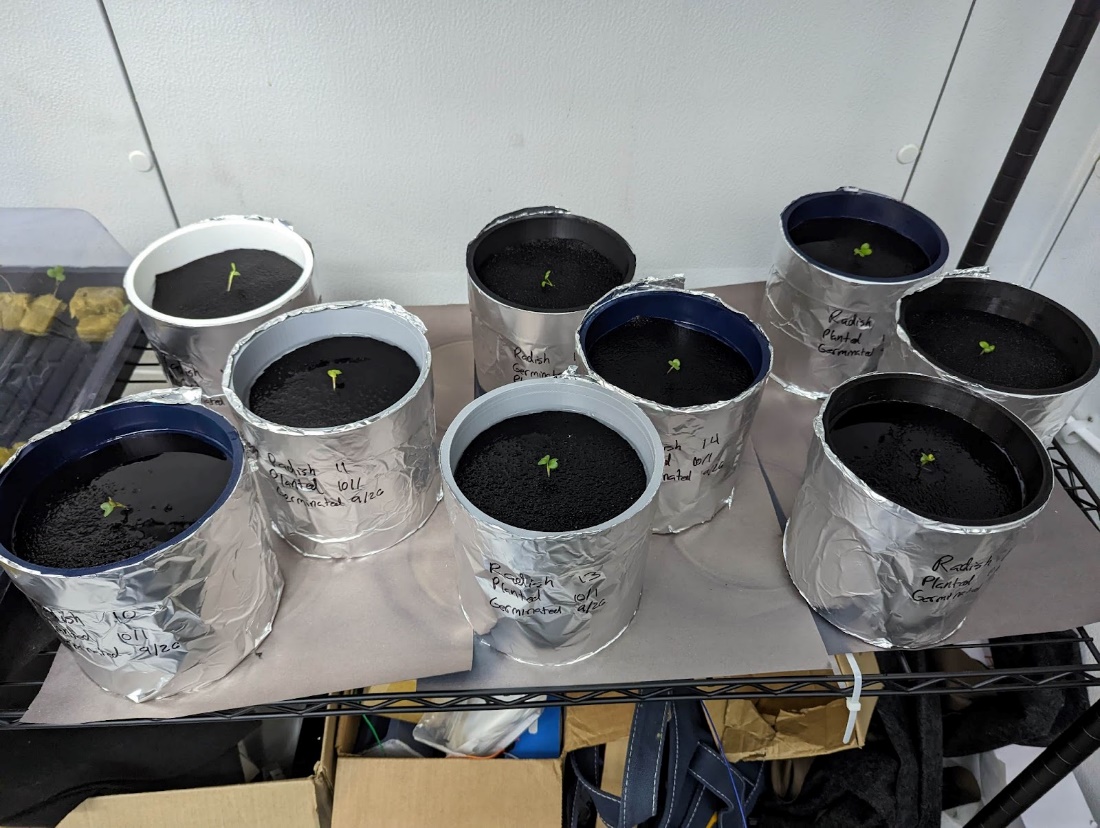


Fig. S1. 9 of the 18 plants grown for the experiment. Plants are labeled with their plant type, planting date and initial volume. A set of grow lights was hung above the plants to assist in growth.


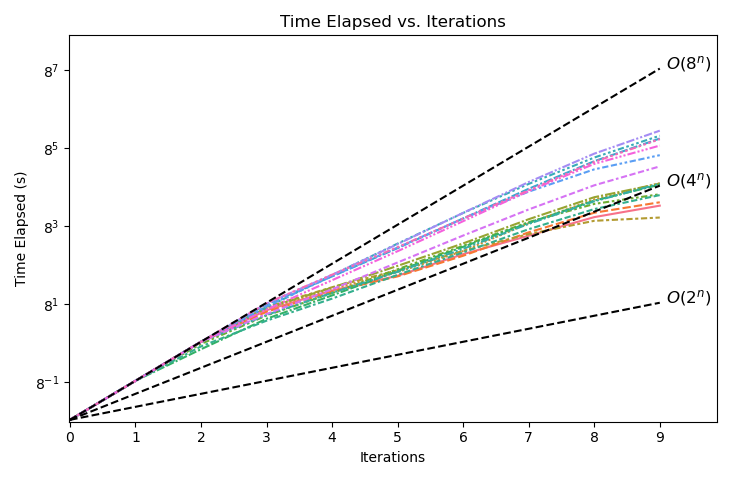


Fig. S2: Time elapsed vs. Space Carving iteration number for our dataset. Timing is plotted on a logarithmic scale.

Table S1: Standard nutrient solution for plant growth. This combination of volume and mass ratios was specifically designed for a 6L container. For some compounds, a pre-mixed water concentrate was not available, so its corresponding solid salt form was used instead. Also, compounds H_3_BO_3_, MnSO_4_·H_2_O, Na_2_MoO_4_·2H_2_O, CuSO_4_·5H_2_O, and ZnSO_4_·7H_2_O were mixed together as a single mix of micronutrients

| **Compound** | **Concentrate Volume (mL)** | **Salt Mass(g)** | **Concentration (g/L)** |
| --- | --- | --- | --- |
| MgSO_4_·7 H_2_O | 44.93 | - | 0.2461 |
| Ca(NO_3_)_2_·4 H_2_O | 51.42 | - | 0.4859 |
| NH_4_NO_3_ | - | 0.69872 | 0.038 |
| KH_2_PO_4_ | 45.00 | - | 0.136 |
| KNO_3_ | - | 7.9553 | 0.442 |
| H_3_BO_3_ | 4.50 | - | 0.0009 |
| MnSO_4_ | 4.50 | - | 0.0008 |
| Na2MoO_4_·2H_2_O | 4.50 | - | 0.00006 |
| CuSO_4_·5H_2_O | 4.50 | - | 0.00009 |
| ZnSO_4_ · 7 H_2_O | 4.50 | - | 0.0006 |
| C_14_H_21_FeN_3_O_10_ | 45.17 | - | 0.01 |
