# Supplementary material for: Toward an Automated System for Nondestructive Estimation of Plant Biomass
Source: Plant Direct. 2025 Mar 19;9(3):e70043. doi: 10.1002/pld3.70043 (PMC11920584; doi:10.1002/pld3.70043)
Supplement: Supplementary file 1 — Data S1. Peer Review [file PLD3-9-e70043-s001.pdf]

Plant Direct
Open Access

Towards an Automated System for Nondestructive Estimation of Plant Biomass

Randall Kliman1, Yuankai Huang2, 4, Ye Zhao3, Yongsheng Chen2*

Decision Letter Round 1:

September 22, 2024

Prof. Yongsheng Chen
Georgia Institute of Technology
School of Civil and Environemental Engineering
Atlanta 30332

RE: Towards an Automated System for Nondestructive Estimation of Plant Biomass

Dear Dr. Chen:

Thank you for submitting to Plant Direct. All required reviews have been returned and we have now finished our evaluation of your manuscript. In light of the reviewers' and editor's comments, further substantial revisions are needed before the paper can be accepted for publication in Plant Direct. These revisions would require additional experimental and analysis work to be acceptable.

Both reviewers requested more complete benchmarking data to ground truth the computational methods. At least 5 different sized known ground truth objectives should be imaged and measured to do regression analysis of validation data, with the ground truth data predicted by the imaged/computed data. While reviewer 1 suggests new algorithms, this is not necessary if the authors can show their pipeline predicts ground truth data as described here.

Otherwise, please follow their advice to make the work more reproducible with good descriptions of hardware and methods, and make other changes to take into account their suggestions as needed.

When uploading the revised version of this article, please be sure to include the following:

-A word document that contains your response to the reviewers. You should respond to each reviewer comment and note the changes made to the manuscript. If you do not agree with a reviewer's comment and choose not to make a suggested revision, please explain why. Please try to provide as complete an answer as possible to each reviewer's criticisms in the "response to reviewers" section.
-A tracked changes document
- A clean version of the latest version of the manuscript

To upload your revision, please click the link below.
https://plantdirect.msubmit.net/cgi-bin/main.plex?el=A2Lr3ga2A4ofl3I5A9ftdm40iK2XAjXNF8W0Gz1zg1AZ

In order to provide as timely a service as possible, we ask that your revision is resubmitted within three months after receipt of this request. If an extension is needed, please send a request, along with a brief explanation, to the editorial office at plantdirect@wiley.com .

Thank you very much for giving us an opportunity to review your work. I look forward to receiving the next version.

Sincerely,
Larry York

-- Reviewer comments:
Reviewer #1:

The authors describe a new method for 3D reconstruction of plant roots. Here are my observations:
1. From section 2, it was not clear what camera was used. From figure 3, it is looking like a vision camera was employed in the experimental setup. Please provide more information about the camera.

2. The authors mentioned that they used robotic arm (Pages 1 and 2), it was not clear as to why a robotic arm is needed. The authors mentioned that they used robotic arm for greatest range of motion to image both root and shoot structures. The robotic arm may not be needed if the authors used two cameras placed on top of the other with sufficient spacing. That way both the 40 images of roots and shoots would be taken without the disadvantages of sacrificing localization accuracy. Does the advantage of having a robotic arm outweigh other methods of imaging?
3. In section 2(C), instead of selecting between SfM, NeRF and space carving, the authors may want to compare the 3D reconstruction method of space carving to that of Inverse Radon transform, since both methods are for silhouettes and probably both methods are susceptible to distortion caused by refraction of various growth media.
4. In section 3, the authors mentioned that their method was validated using a single 3D printed root structure and comparing its estimated volume to the water displacement. I believe that comparing using just a single 3D printed sample may not help the reader who may want to adopt the method described in the paper. The authors need to 3D print several 3D roots and make a correlation plot of the estimated volume and the real volume from the water displacement. I would also recommend the authors also 3D print simple geometric shapes such as cube, tetrahedron, cone and a sphere and discuss which shape causes maximum discrepancy in volume estimation. To validate the method further, it is also important to make correlation plot of real roots, as synthetic root structures are never like real plant roots. The method may be non-destructive, but the validation need not be.
5. Figure 7 is not informative enough. Please also add a plant root image with and without distortion due to refraction. According to the imaging setup, the refraction happens at two stages. The first stage occurs between the air and the outer surface of the pot. The second stage occurs between the inner surface of the pot and the nutrient solution. Please also discuss how the distortion affects the estimation of root volume. How much does an object of known size shrink due to refraction?
6. The title mentioned that the authors worked on plant biomass estimation. The paper also mentioned that the imaging system takes 40 shoot and 40 root images. But the shoot images were not shown/discussed except in the imaging system. The idea of non-destructive estimation only applies to root imaging. Maybe imaging shoot system is advantageous in this study because the shoot images are not distorted due to refraction and hence these images make good talking points as to how the distortion adversely affects root volume estimation.
7. Complex roots have "poor" reconstruction results due to occlusion, which the authors did not discuss upon. This is one of the reasons why some research studies use X-Ray CT scans to compute 3D-Reconstructions as a non-destructive method to estimate root volumes. Since, the authors use a monochromatic/RGB camera to image the root system, the problem of occlusion

may not be solved. There may be a tradeoff that authors may need to take. If the authors are interested in accuracy, then X-Ray CT imaging system may be useful, which may not be cost effective.
8. The Table 1 showed the estimated volumes. Of these which ones are "good" reconstructions and which ones are "poor" reconstructions? The volume estimates for Roots #17 and #18 are not shown.
9. If possible, add a sub plot to figure 8 that shows the scatter plot of number of iterations needed to converge versus either the estimated volume or days elapsed from Table 1. This may show some insight on how much time the reconstruction method takes from simple to complex roots. On average how much time did it take for the authors to image one plant and how much time did it take to process and generate a 3D reconstruction?
10. The authors described the 3D reconstruction method but did not discuss how the volume is computed from the 3D model. Understanding how root volume is computed may also shed some light on low accuracy of computed volumes as described in section 4.
11. Please provide all the models used for 3D printing in the supplemental data.

Reviewer #2:

The paper presents an innovative approach to analyzing the growth of lettuce plants in hydroponic systems and discusses the creation of an automated system for the estimation of plant root and shoot biomass. While the study demonstrates potential, several critical issues need to be addressed to enhance clarity, and coherence. Below are major revision suggestions:
• The abstract lacks a clear statement of the specific problem being addressed. Begin with a sentence that clearly outlines the challenge or gap in current methods.
• Lack of novelty. Explicitly state the main objective of the study and summarize the key contributions, including the novelty of using a robotic arm and the specific imaging and processing techniques developed.
• Include a detailed description of the experimental setup, including the hydroponic system, plant growth conditions, and imaging equipment. An overall diagram may be helpful for better understanding.
• Explain the reasons behind the resolution limitation to 0.27mm and its impact on the quality of the 3D reconstructions.
• Comparison with Existing Methods: Discuss how your results compare with those obtained using traditional methods and recent advancements.

Plant Direct
Open Access

Decision Letter Round 2:

January 9, 2025
Prof. Yongsheng Chen
Georgia Institute of Technology
School of Civil and Environemental Engineering
Atlanta, Georgia 30332

MSID: 2024-01444-TW1
MS TITLE: Towards an Automated System for Nondestructive Estimation of Plant Biomass

Dear Dr. Yongsheng Chen:

I am pleased to inform you that your manuscript "Towards an Automated System for Nondestructive Estimation of Plant Biomass" has been accepted for publication in Plant Direct. As a root biologist, I greatly appreciate your contributions to the field.

Your article will appear online in the next available issue of Plant Direct. To ensure your article gets published as quickly as possible, please pay attention to the steps detailed below. We have found that most of the delays happen at this stage, especially at the payment stage, so please respond as quickly as possible when prompted.

License Agreement: Once your article has been accepted it will move to Production and undergo admin and file checking - you may receive an email with any queries we have at this stage. When all required items are received by the publisher and queries resolved, the corresponding author will receive an email from Wiley's Author Services system which will ask them to log on at https://authorservices.wiley.com/bauthor and will present them with the appropriate license for completion. Your article cannot be published until both the signed license agreement and payment of the article fee have been received.

Payment of the Open Access Article Publication Fee: All articles published in Plant Direct are fully open access: immediately and freely available to read, download and share. Plant Direct charges a publication fee to cover the publication costs. The corresponding author for this manuscript should have already received a quote from the payments team (cs-openaccess@wiley.com) with the estimated article publication fee; please email cs-openaccess@wiley.com if this has not been received. The corresponding author should log on

to the Wiley Author Services site, where the publication fee can be paid by credit card or an invoice. Pro Forma can also be requested. Payment of the publication charge must be received before the article will be published online.

Proofs: You will have the opportunity to look over your paper once more when you receive the author proofs for your article. The proofs will be with you in approximately two weeks. Please note that, in addition to publishing reviewer comments, the author's responses to review comments will also be published alongside the final version of the paper. If you would not like the author's responses to be published, please contact the editorial office at plantdirect@wiley.com .

Promotion of your article: You can help your research get the attention it deserves! Check out Wiley's free Promotion Guide for best-practice recommendations for promoting your work at www.wileyauthors.com/eeo/guide. And learn more about Wiley Editing Services which offers professional video, design, and writing services to create shareable video abstracts, infographics, conference posters, lay summaries, and research news stories for your research at www.wileyauthors.com/eeo/promotion.

Thank you again for your contribution to Plant Direct. If you have any questions, feel free to contact the editorial office at plantdirect@wiley.com .

Sincerely,

Larry York

EIC Plant Direct

Response to Reviewer:

We thank the valuable and insightful comments from reviewers. All these comments have been thoroughly addressed throughout the manuscript using track/change.

Reviewer(s)' Comments to Author:

Reviewer #1:

The authors describe a new method for 3D reconstruction of plant roots. Here are my observations:

1. From section 2, it was not clear what camera was used. From figure 3, it is looking like a vision camera was employed in the experimental setup. Please provide more information about the camera.

Response: We appreciate your thoughtful comment about which camera system was used. The camera system used in the setup was a Raspberry Pi HQ camera which was connected via the Raspberry Pi's CSI interface. This camera boasts a 12.3-megapixel sensor, an adjustable focus, and a considerable FOV. Compared to other cameras on the market at the time, this one was the best quality to price camera that was easily interoperable with the Raspberry Pi we used for computation and control. Additionally, it provided an easy software interface. A link to the camera system is provided here:

https://www.raspberrypi.com/products/raspberry-pi-high-quality-camera/.

We have revised the manuscript to clarify this information. The revised text as it appears in the manuscript in red color as follows:

The camera system used is a Raspberry Pi High Quality Camera which has a 12.3 megapixel sensor and can output RAW images. This camera was chosen for its high resolution, adjustable focus, and reasonable field of view. Additionally, the software interface and support for this camera were quite simple and easy to use. The camera system is connected to the Raspberry Pi using a Camera Serial Interface (CSI) cable and communicates with the Raspberry Pi over the CSI interfacing protocol.

2. The authors mentioned that they used robotic arm (Pages 1 and 2), it was not clear as to why a robotic arm is needed. The authors mentioned that they used robotic arm for greatest range of motion to image both root and shoot structures. The robotic arm may not be needed if the authors used two cameras placed on top of the other with sufficient spacing. That way both the 40 images of roots and shoots would be taken without the disadvantages of sacrificing localization accuracy. Does the advantage of having a robotic arm outweigh other methods of imaging?

Response: We appreciate your insightful comment regarding the use of a robotic arm in our imaging system. The primary reason for utilizing the robotic arm is to efficiently capture images of both the root and shoot structures with a single camera. The robotic arm allows us to reposition the camera between imaging the roots and shoots without manual intervention or the need for multiple fixed cameras.

While using two fixed cameras is a viable alternative, the robotic arm simplifies the imaging setup by reducing the number of cameras and associated calibration requirements. It enables us to adjust the camera's position and angle to accommodate different plant sizes and growth stages, ensuring consistent image quality throughout the experiment. This flexibility is particularly beneficial as plants grow and change shape over time.

We have revised the manuscript to clarify the primary reason for using the robotic arm and to address your concern. The revised text as it appears in the manuscript in red color as follows:

The primary reason for using the robotic arm is to facilitate the imaging of both root and shoot structures efficiently. The robotic arm allows us to reposition the camera to capture images from optimal angles for both the roots and the shoots without manual adjustment or multiple cameras. This flexibility simplifies the imaging process and ensures consistent image quality throughout the experiment.

3. In section 2(C), instead of selecting between SfM, NeRF and space carving, the authors may want to compare the 3D reconstruction method of space carving to that of Inverse Radon transform, since both methods are for silhouettes and probably both methods are susceptible to distortion caused by refraction of various growth media.

Response: We appreciate your insightful suggestion to compare the 3D reconstruction method of space carving with the Inverse Radon Transform (IRT). Both methods indeed utilize silhouettes for reconstruction and are susceptible to distortion caused by refraction in various growth media. Including this comparison enhances the discussion of our methodology and clarifies our choice of space carving over IRT.

The IRT is a mathematical technique commonly used in computed tomography (CT) for reconstructing cross-sectional images from projection data. It is highly effective when projections are taken at precise, uniformly distributed angles around the object and when the imaging system can capture attenuation coefficients directly, as in CT scanners.

However, in our application, several factors influenced our decision to use space carving over IRT:

1. IRT assumes parallel beam projections, which is suitable for CT scans but not for optical imaging systems like ours that involve perspective projections due to the camera's finite distance from the object.
2. Plant roots present complex, non-uniform, and concave geometries that are better handled by space carving methods designed for arbitrary shapes, whereas IRT is more suitable for reconstructing objects with uniform attenuation properties.
3. Both methods are susceptible to refraction-induced distortions. However, space carving allows for integration with raytracing techniques to correct for these distortions, as we have implemented in our method.
4. Space carving can work effectively with silhouettes extracted from images, even when the data is incomplete or noisy. IRT generally requires high-quality projection data and precise control over imaging parameters, which is challenging in our setup.

Based on these considerations, we believe space carving is more appropriate for our specific application. We have revised Section II(C) of the manuscript to include a discussion comparing space carving with the Inverse Radon Transform and to justify our methodological choice. The revised text as it appears in the manuscript in red color as follows:

The Inverse Radon Transform is a foundational technique in computed tomography (CT) for reconstructing an object from its projections. It assumes parallel beam projections and requires precise control over projection angles and imaging conditions. However, applying IRT to our context poses several challenges, since plant root systems have complex, non-uniform shapes with concavities that are difficult to reconstruct accurately using IRT, which is better suited for objects with uniform properties. Additionally, IRT requires high-quality, evenly distributed projection data. In our setup, achieving such precise control is challenging, and the silhouettes may contain noise due to imaging conditions. Thus, because SfM, NeRF and IRT are unavailable to use, Space Carving is determined to be the most suitable method for 3D reconstruction.

4. In section 3, the authors mentioned that their method was validated using a single 3D printed root structure and comparing its estimated volume to the water displacement. I believe that comparing using just a single 3D printed sample may not help the reader who may want to adopt the method described in the paper. The authors need to 3D print several 3D roots and make a correlation plot of the estimated volume and the real volume from the water displacement. I would also recommend the authors also 3D print simple geometric shapes such as cube, tetrahedron, cone and a sphere and discuss which shape causes maximum discrepancy in volume estimation. To validate the method further, it is also important to make correlation plot of real roots, as synthetic root structures are never like real plant roots. The method may be non-destructive, but the validation need not be.

Response: We appreciate the reviewer's thoughtful comment regarding validating our 3D reconstruction method using multiple 3D-printed objects. We agree that a more extensive validation with various shapes and sizes would strengthen the reliability and applicability of our method.

In our initial development phase, we conducted preliminary validation experiments using several 3D-printed objects, including simple geometric shapes like cubes of different sizes. These experiments aimed to assess the accuracy of our algorithm across objects with known volumes. The algorithm performed well, reconstructing these shapes with an average accuracy of approximately 89%.

To provide validation within the scope of our current study, we revisited our existing dataset from the longitudinal study involving 18 radish plants. Although our method is nondestructive, we performed destructive validation by harvesting the plants after imaging. We then measured the actual volumes of the roots using water displacement and compared these values with the estimated volumes from our 3D reconstructions. This allowed us to create a correlation between the estimated and actual volumes across multiple samples of real plant roots. The results demonstrated a reasonable correlation between the estimated volumes and the actual volumes, supporting the effectiveness of our method in estimating plant biomass nondestructively. We have included these findings in the revised manuscript, along with a discussion of the limitations and potential sources of error.

We acknowledge that additional validation using a variety of 3D-printed objects and more detailed correlation analyses would further enhance our study. We plan to incorporate such comprehensive validation in future work, systematically assessing the accuracy of our method across various shapes and complexities. The revised text as it appears in the manuscript in red color as follows:

First, we sought to validate that the model could accurately reconstruct plant volume. This was done in two ways. The first validation was done by printing a set of cubes in PLA ranging from 50x50x50mm(125ml) to 125x125x125mm(1953.125ml). The algorithm performed well, reconstructing each to a roughly 89% accuracy. We also validated our model by 3D printing synthetic root structures in solid PLA and comparing their measured volume using water displacement with their calculated volume using our method. We measured the synthetic root structure to be 8ml and calculated the volume to be 7.13ml. These measurements are very close, and the error can be attributed to poor camera pose estimation as well as aggressive voxel culling.

5. Figure 7 is not informative enough. Please also add a plant root image with and without distortion due to refraction. According to the imaging setup, the refraction happens at two stages. The first stage occurs between the air and the outer surface of the pot. The second stage occurs

between the inner surface of the pot and the nutrient solution. Please also discuss how the distortion affects the estimation of root volume. How much does an object of known size shrink due to refraction?

Response: Thank you for your valuable feedback on Figure 7 and the discussion of optical distortion due to refraction in our imaging setup. We agree that providing images of the plant root with and without distortion and quantifying how distortion affects the estimation of root volume would enhance the reader's understanding of the impact of refraction.

Unfortunately, due to constraints in our experimental setup and the unavailability of additional data, we are unable to provide images of the plant root without distortion or quantify the exact extent of shrinkage caused by refraction at this time.

However, to address your concerns, we have expanded the text in Section II.E to:

- Explain the two stages of refraction occurring in our system: from air to the outer surface of the acrylic canister and from the inner surface of the canister to the nutrient solution.
- Discuss qualitatively how distortion affects the estimation of root volume.
- Emphasize the importance of our raytracing correction method in mitigating these distortion effects.

We believe that these additions provide a clearer understanding of the challenges posed by optical distortion and how our method addresses them, even without the specific quantitative data. We hope this satisfactorily addresses your comment. The revised text as it appears in the manuscript in red color as follows:

One major challenge in the imaging of hydroponic systems is the distortion caused by refraction due to various growth media. In our experiment, we grow plants hydroponically in cylindrical acrylic canisters, which introduces significant optical distortion due to refraction at the interfaces between different media. There are two stages of refraction in our system: 1. Light rays traveling from the air into the acrylic canister refract at the outer surface because of the difference in refractive indices between air ($n \approx 1.00$) and acrylic ($n \approx 1.49$); 2. Light rays refract again at the inner surface of the canister when passing from acrylic into the nutrient solution (water with dissolved nutrients, $n \approx 1.33$). These two stages of refraction cause significant optical distortion, making objects inside the canister appear compressed or shifted from their true positions. This distortion affects both the apparent size and shape of the root structures, leading to inaccuracies in camera pose estimation and 3D reconstruction if not properly corrected.

To address this issue, we developed a raytracing algorithm based on previous research and used the previously calculated camera extrinsic for each view. We cast 480,000 (800x600) rays from the camera at a simulated cylinder with the same dimensions as the growth canister, and after these rays collided and refracted on both the inside and outside surfaces of the canister, a set of points and their resulting ray vectors are generated on the inside surface of the cylinder. Figure 7 visualizes this process. Optical distortion affects the estimation of root volume by altering the apparent dimensions of the root structures. The refraction causes the roots to appear smaller or differently shaped than they actually are, which can lead to underestimation or overestimation of the root volume when reconstructing the 3D model.

Without correction, these distortions can significantly compromise the accuracy of biomass estimations.

For each voxel at every view, we cast these rays towards virtual voxels and checked for intersection. Using a 3x800x600 ray tensor with a 3D vector as the first dimension and the remaining dimensions as the shape of our images, we created a binary mask indicating whether the ray intersected a voxel. This mask can be applied to our original silhouette using a bitwise AND operation to determine the proportion of each voxel that is visible, allowing for a more accurate estimation of voxel membership in the model. By applying this correction, we effectively mitigate the distortion caused by refraction, aligning the apparent positions of the root structures with their true positions. This correction improves the accuracy of camera pose estimation and the subsequent 3D reconstruction of the root system. To speed up this process, we developed a raytracing shader which was applied to an image for each voxel and was parallelized on a GPU using the python package CuPy (Okuta et al. 2017).

6. The title mentioned that the authors worked on plant biomass estimation. The paper also mentioned that the imaging system takes 40 shoot and 40 root images. But the shoot images were not shown/discussed except in the imaging system. The idea of non-destructive estimation only applies to root imaging. Maybe imaging shoot system is advantageous in this study because the shoot images are not distorted due to refraction and hence these images make good talking points as to how the distortion adversely affects root volume estimation.

Response: Thank you for your thoughtful feedback regarding the inclusion of shoot images and their potential role in highlighting the impact of optical distortion on root volume estimation. We acknowledge that the manuscript did not sufficiently discuss the shoot images or their analysis, which may have led to confusion given that the title mentions plant biomass estimation and the imaging system captures both root and shoot images.

The primary focus of our study was on developing and validating methods for nondestructive estimation of **root biomass**, which presents significant challenges due to optical distortions from refraction and occlusions within complex root structures in hydroponic systems. While we did capture images of the shoot system, the analysis of these images was beyond the scope of the current study. Our intention was to address the more complex problem of root imaging and reconstruction, where existing methods are limited.

We agree that imaging the shoot system offers advantages, particularly because it is not affected by optical distortions due to refraction. Including a discussion of the shoot imaging could provide valuable context and contrast to the root imaging results. To address your comment, we have revised the manuscript to:

- Clarify the focus of our study on root biomass estimation in the Introduction and throughout the text.
- Acknowledge the collection of shoot images and briefly discuss how the lack of optical distortion in shoot imaging contrasts with the challenges faced in root imaging.

- Explain why the analysis of shoot images was not included in detail in this paper, to maintain a focused scope on the root system.

We believe these revisions enhance the clarity of our manuscript and address your concern by providing context for the inclusion of shoot images in our imaging process, even if detailed analysis is not presented. The revised text as it appears in the manuscript in red color as follows:

The main objective of this study is to develop an automated, low-cost system for nondestructive estimation of plant root biomass throughout the plant's lifecycle in hydroponic systems. We achieve this by utilizing a robotic arm and turntable to capture images from multiple angles, allowing for comprehensive 3D reconstruction. Our key contributions include the novel use of a robotic arm for flexible imaging, which allows dynamic positioning of the camera to capture detailed images of root structures without the need for multiple fixed cameras or manual repositioning. Additionally, we develop customized 3D reconstruction techniques, including a voxel-based volumetric reconstruction method using space carving and a raytracing-based optical correction algorithm to address optical distortion due to refraction in the growth medium. By integrating root and shoot imaging, our approach provides a more comprehensive estimation of plant biomass than methods focusing solely on one system. Our system significantly improves the resolution at which these models can be generated and allows for biomass estimations with sub-millimeter resolution.

While we collected shoot images during our experiments, the analysis and reconstruction of the shoot system were considered beyond the scope of this research. Our primary goal was to address the significant challenges in root biomass estimation. Future work may involve analyzing the shoot images to provide a comprehensive assessment of total plant biomass and to further compare the differences in imaging conditions between roots and shoots.

7. Complex roots have "poor" reconstruction results due to occlusion, which the authors did not discuss upon. This is one of the reasons why some research studies use X-Ray CT scans to compute 3D-Reconstructions as a non-destructive method to estimate root volumes. Since, the authors use a monochromatic/RGB camera to image the root system, the problem of occlusion may not be solved. There may be a tradeoff that authors may need to take. If the authors are interested in accuracy, then X-Ray CT imaging system may be useful, which may not be cost effective.

Response: We appreciate your insightful comment regarding the challenges of occlusion in reconstructing complex root systems using optical imaging methods. We acknowledge that occlusion can lead to "poor" reconstruction results, especially in dense root structures where parts of the roots block others from the camera's view. This is a known limitation of using monochromatic/RGB cameras for imaging plant roots.

While X-ray computed tomography (CT) scans can effectively overcome occlusion and provide high-accuracy 3D reconstructions, they come with significant drawbacks such as high cost, limited

accessibility, and potential safety concerns associated with radiation exposure. We aimed to develop a low-cost, accessible method for nondestructive estimation of plant biomass that can be widely adopted in both research and industry settings.

To address the issue of occlusion in our optical imaging approach, we have discussed this limitation in the manuscript and acknowledged the trade-offs between accuracy and practicality. We have also suggested potential future improvements, such as increasing the number of imaging angles and exploring advanced reconstruction algorithms to mitigate the effects of occlusion.

We have added a brief discussion in the manuscript to acknowledge these points. The revised text as it appears in the manuscript in red color as follows:

An inherent limitation of our optical imaging method is the issue of occlusion, particularly when dealing with complex and dense root systems. Occlusion occurs when parts of the root structure block others from the camera's view, leading to incomplete or less accurate reconstructions. While X-ray computed tomography (CT) scans can effectively overcome occlusion and provide high-accuracy 3D reconstructions, they are expensive, less accessible, and involve potential safety concerns due to radiation exposure. Our approach prioritizes practicality and cost-effectiveness, aiming to provide a nondestructive biomass estimation technique that is accessible for broader applications. Future work may focus on mitigating occlusion effects by increasing the number of imaging angles or incorporating advanced computational methods to improve reconstruction accuracy despite occlusions.

8. The Table 1 showed the estimated volumes. Of these which ones are "good" reconstructions and which ones are "poor" reconstructions? The volume estimates for Roots #17 and #18 are not shown.

Response: Thank you for pointing out the missing data for roots 17 and 18. The 3D reconstructions for both of these plants failed due to imaging difficulties and they were pulled out of the water and weighed before this fact was known. This is good to make note of though, and we have added a brief note to acknowledge this point. The revised text as it appears in the manuscript in red color as follows:

3D reconstruction efforts on Roots 17 and 18 failed, thus there are no estimates of volume or density for these measurements.

9. If possible, add a sub plot to figure 8 that shows the scatter plot of number of iterations needed to converge versus either the estimated volume or days elapsed from Table 1. This may show some insight on how much time the reconstruction method takes from simple to complex

roots. On average how much time did it take for the authors to image one plant and how much time did it take to process and generate a 3D reconstruction?

Response: We appreciate your note about adding a subplot to discuss the timing of the algorithm. We have added the following plot with the data that is available as well as an explanation of the timing constraints. The revised text as it appears in the manuscript in red color as follows:

Figure S2 shows how much time each iteration adds to the space carving for the 18 plants grown. The graph shows that the data follows a roughly exponential scale, reaching a timing complexity of around 5^n where n is the number of iterations. Thus, it made sense to limit the voxel size, as an additional iteration step would increase our resolution to approximately 0.13 mm but would lead to a 5x increase in computational time. Additionally, more mature root systems cannot benefit from the computational gains of the octree data structure due to their spread-out nature and higher volume. For example, root structures in the early stages of development can be processed to 0.27mm in only 25 minutes, but more mature root structures take nearly 24 hours to process. Increasing the resolution to 0.13mm would increase processing time to 125 minutes for a small root system and up to 5 days for a mature root system.

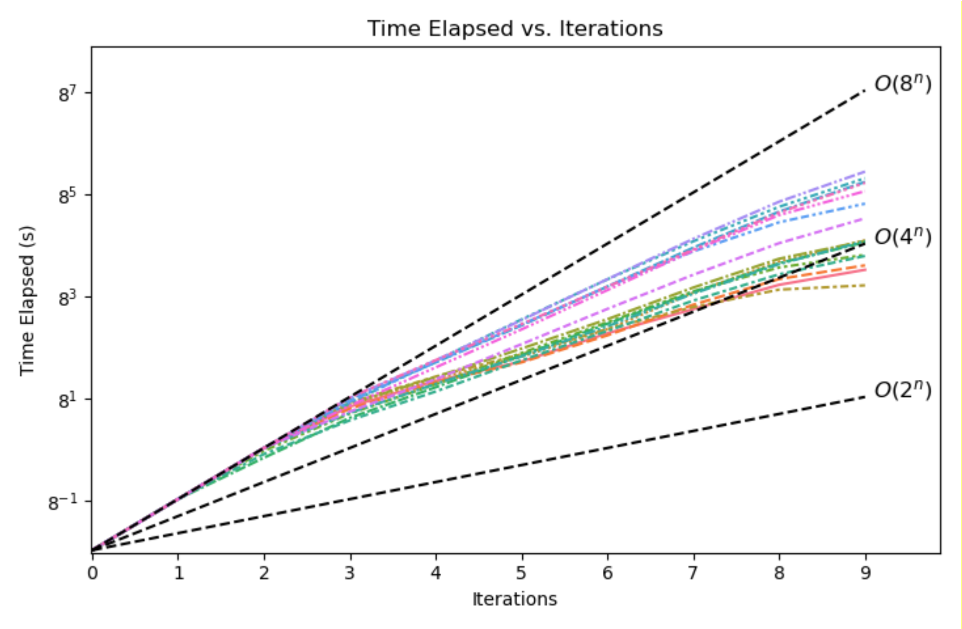

Fig. S2: Time elapsed vs. Space Carving iteration number for our dataset. Timing is plotted on a logarithmic scale.

10. The authors described the 3D reconstruction method but did not discuss how the volume is computed from the 3D model. Understanding how root volume is computed may also shed some light on low accuracy of computed volumes as described in section 4.

Response: Thank you for pointing out the omission regarding the computation of volume from the 3D model. We agree that a detailed explanation of how the volume is calculated from the reconstructed 3D model is crucial for understanding our methodology and the factors contributing to the low accuracy observed in Section 4.

In response, we have revised the manuscript to include a detailed description of the volume computation process from the 3D model. We have added this information to Section II.F (Octree Space Carving) and expanded the discussion in Section IV (Experimental Results) and Section V (Discussion) to shed light on how the method of volume calculation affects the accuracy of the computed volumes. The revised text as it appears in the manuscript in red color as follows:

After reconstructing the 3D model of the plant roots using the octree-based space carving method, we compute the volume by summing the volumes of all occupied voxels in the final octree structure. Each voxel represents a small cube in 3D space, and its volume is determined by the cube of its side length. The total root volume V_{total} is calculated as:

$$Vtotal=N\times s^3$$

Where N is the total number of occupied voxels, and s is the side length of a voxel, determined by the resolution of the octree. This method allows us to estimate the volume of the root system based on the spatial occupancy of the voxels in the reconstructed model.

11. Please provide all the models used for 3D printing in the supplemental data.

Response: Thank you for your valuable suggestion. We agree that providing the 3D models used for printing will be beneficial for readers interested in replicating our experiments or further validating our methods. We have included the STL files for 3D models in the Supplemental Information (SI) section of the manuscript.

Regarding the code used to generate these models, we would like to keep it unpublished due to confidentiality reasons. However, by providing the STL files, we ensure that interested readers have access to the necessary resources to replicate the 3D printing and validation processes described in our study.

Reviewer #2:

The paper presents an innovative approach to analyzing the growth of lettuce plants in hydroponic systems and discusses the creation of an automated system for the estimation of plant root and shoot biomass. While the study demonstrates potential, several critical issues need to be addressed to enhance clarity, and coherence. Below are major revision suggestions:

1. The abstract lacks a clear statement of the specific problem being addressed. Begin with a sentence that clearly outlines the challenge or gap in current methods.

Response: Thank you for your insightful feedback regarding the clarity of our abstract. We agree that it lacked an explicit statement of the specific problem being addressed. To enhance clarity and immediately inform the reader of the challenge our work tackles, we have revised the abstract, to begin with a clear sentence outlining the gap in current methods for nondestructive estimation of plant biomass. This revision aims to provide a concise introduction to the problem and set the context for our research. The revised text as it appears in the manuscript in red color as follows:

Accurate and nondestructive estimation of plant biomass is crucial for optimizing plant productivity, but existing methods are often expensive and require complex experimental setups. To address this challenge, we developed an automated system for estimating plant root and shoot biomass over the plant's lifecycle in hydroponic systems. This system employs a robotic arm and turntable to capture 40 images at equidistant angles around a hydroponically grown lettuce plant. These images are then processed into silhouettes and used in voxel-based volumetric 3D reconstruction to produce detailed 3D models. We utilized a space carving method along with a raytracing-based optical correction technique to create high-accuracy reconstructions. Analysis of these models demonstrates that our system accurately reconstructs the plant root structure and provides precise measurements of root volume, which can be calibrated to indicate biomass.

2. Lack of novelty. Explicitly state the main objective of the study and summarize the key contributions, including the. novelty of using a robotic arm and the specific imaging and processing techniques developed.

Response: Thank you for your valuable feedback regarding the need to explicitly state the main objective and highlight the novelty of our study. We agree that the introduction can be strengthened by clearly articulating our research objective and summarizing our key

contributions, including the innovative use of a robotic arm and the specific imaging and processing techniques we developed.

To address this, we have revised the Introduction section to:

- Clearly state the main objective of our study.

Summarize the key contributions and novel aspects of our work, emphasizing the use of a robotic arm for flexible imaging and our customized 3D reconstruction methods. The revised text as it appears in the manuscript in red color as follows:

Our method is one of few which focus exclusively on Root Structure Architecture(RSA), which addresses these challenges by providing a novel approach that combines flexible imaging and advanced reconstruction techniques to estimate plant biomass nondestructively. The main objective of this study is to develop an automated, low-cost system for nondestructive estimation of plant root and shoot biomass throughout the plant's lifecycle in hydroponic systems. We achieve this by utilizing a robotic arm and turntable to capture images from multiple angles, allowing for comprehensive 3D reconstruction. Our key contributions include the novel use of a robotic arm for flexible imaging, which allows dynamic positioning of the camera to capture detailed images of both root and shoot structures without the need for multiple fixed cameras or manual repositioning. Additionally, we develop customized 3D reconstruction techniques, including a voxel-based volumetric reconstruction method using space carving and a raytracing-based optical correction algorithm to address optical distortion due to refraction in the growth medium. By integrating root and shoot imaging, our approach provides a more comprehensive estimation of plant biomass than methods focusing solely on one system. Our system significantly improves the resolution at which these models can be generated and allows for biomass estimations with sub-millimeter resolution.

3. Include a detailed description of the experimental setup, including the hydroponic system, plant growth conditions, and imaging equipment. An overall diagram may be helpful for better understanding.

Response: We appreciate your valuable feedback about the quality of the explanation of the experimental setup. While many of the individual parts of the experimental setup were described, more detail can be added to discuss the growing conditions of the plants. Additionally, a picture of the growing conditions and has been added. The revised text as it appears in the manuscript:

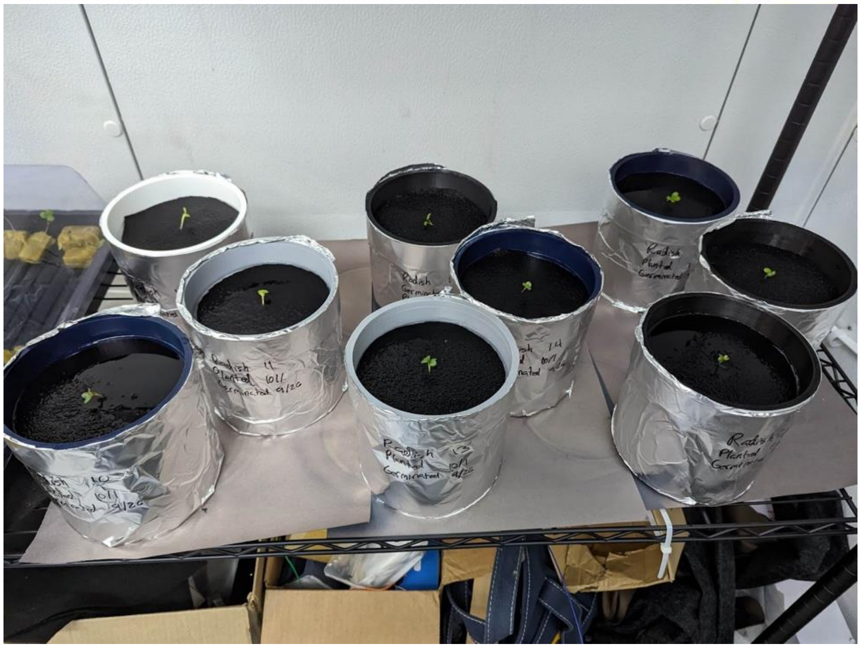

Fig. S1. 9 of the 18 plants grown for the experiment. Plants are labeled with their plant type, planting date and initial volume. A set of grow lights was hung above the plants to assist in growth.

Table S1: Standard nutrient solution for plant growth. This set of volume and mass ratios were designed for a 6L container. For some compounds, a pre-mixed water concentrate was not available, so its corresponding solid salt form was used instead. Also, compounds H_3BO_3, $MnSO_4{\cdot}H_2O$, $Na_2MoO_4{\cdot}2H_2O$, $CuSO_4{\cdot}5H_2O$, and $ZnSO_4{\cdot}7H_2O$ were mixed together as a single mix of micronutrients

Compound	Concentrate Volume (mL)	Salt Mass(g)	Concentration (g/L)
$MgSO_4{\cdot}7\ H_2O$	44.93	-	0.2461
$Ca(NO_3)_2{\cdot}4\ H_2O$	51.42	-	0.4859
NH_4NO_3	-	0.69872	0.038
KH_2PO_4	45.00	-	0.136
KNO_3	-	7.9553	0.442
H_3BO_3	4.50	-	0.0009
$MnSO_4$	4.50	-	0.0008
$Na2MoO_4{\cdot}2H_2O$	4.50	-	0.00006
$CuSO_4{\cdot}5H_2O$	4.50	-	0.00009
$ZnSO_4 \cdot 7\ H_2O$	4.50	-	0.0006
$C_{14}H_{21}FeN_3O_{10}$	45.17	-	0.01

Plants were first germinated for an of 7 days in a rock-wool medium before being removed from the medium and transferred to the canisters. Plants were placed in the net pot and positioned in the center of the canister using two pieces of felt for support. A hole was cut in the center of the circular felt pieces where the plant's roots were carefully positioned through the

hole with the roots on the bottom side of the felt and the shoot on the top side of the felt. The canisters were filled with a nutrient solution and the net pot was placed on top of the canister. The canisters were then placed on a shelf unit which contained a grow light directly above them. The grow lights were set on a timer to be on for 16 hours during the day and off for 8 hours at night. Tin foil was wrapped around the canisters to mitigate algae production. At 5 day intervals, a set of 3 plant canisters were removed from the shelf and placed onto the turntable and imaged using the camera technique. After imaging, the plants were cut to separate root and shoots and their respective wet weights measured. Next, the plants were dried in an oven for 2 days after which their dry masses were measured. Lastly, the pictures taken by the imaging system were sent through the 3D reconstruction algorithm to determine the root volumes. Tin foil was wrapped around the canisters to mitigate algae production. At 5 day intervals, a set of 3 plant canisters were removed from the shelf and placed onto the turntable and imaged using the camera technique. After imaging, the plants were cut to separate root and shoots and their respective wet weights were measured. Next, the plants were dried in an oven for 2 days after which their dry masses were measured. Lastly, the pictures taken by the imaging system were sent through the 3D reconstruction algorithm to determine the root volumes.

4. Explain the reasons behind the resolution limitation to 0.27mm and its impact on the quality of the 3D reconstructions.

Response: Thank you for your insightful comment regarding the resolution limitation to 0.27 mm in our 3D reconstructions. We acknowledge that the manuscript did not sufficiently explain the reasons behind this limitation and its impact on the quality of the reconstructions. We have now revised the manuscript to include an explanation in the "Experimental Methods" section.

The primary reason for limiting the voxel resolution to 0.27 mm was due to computational constraints. Higher resolutions require significantly more memory and processing power, which exceeded the capacity of our available hardware. By selecting a voxel size of 0.27 mm, we aimed to balance achieving sufficient detail in the reconstructions with manageable computation times.

This resolution impacts the quality of the 3D reconstructions by potentially omitting finer root structures, such as root hairs and very thin lateral roots. Consequently, some small features of the root architecture might not be accurately represented, and the estimated volumes may be slightly lower than the actual volumes. Despite this limitation, the chosen resolution was adequate to capture the main structure of the roots and provide useful biomass estimations. The revised text as it appears in the manuscript in red color as follows:

To process the image sets efficiently, we limited the voxel resolution in our 3D reconstruction algorithm to approximately 0.27 mm. This resolution was selected due to computational constraints, as higher resolutions would require significantly more memory and processing time than was available on our hardware. While a finer voxel size could capture more detailed features of the root structures—such as thin roots and root hairs—the chosen resolution provides a balance between reconstruction quality and computational feasibility.

5. Comparison with Existing Methods: Discuss how your results compare with those obtained using traditional methods and recent advancements.

Response: Thank you for your valuable suggestion. We agree that discussing how our results compare with traditional methods and recent advancements will enhance the understanding of our work's significance. We have added a comparison in the Discussion section, highlighting the advantages and limitations of our method relative to existing techniques. The revised text as it appears in the manuscript in red color as follows:

Compared to traditional methods for plant biomass estimation, which often involve destructive sampling and labor-intensive processes, our nondestructive imaging approach provides a practical alternative with reasonable accuracy. While advanced imaging techniques like X-ray CT and MRI offer high-resolution reconstructions and can mitigate issues like occlusion, they are costly and less accessible for widespread use. Recent optical imaging methods have improved nondestructive analysis but still face challenges with complex root structures and optical distortions. Our method strikes a balance between cost, accessibility, and performance, offering improvements over traditional destructive techniques and serving as a feasible option compared to expensive imaging technologies.
